# Dopamine–endocannabinoid interactions mediate spike-timing-dependent potentiation in the striatum

**DOI:** 10.1038/s41467-018-06409-5

**Published:** 2018-10-08

**Authors:** Hao Xu, Sylvie Perez, Amandine Cornil, Bérangère Detraux, Ilya Prokin, Yihui Cui, Bertrand Degos, Hugues Berry, Alban de Kerchove d’Exaerde, Laurent Venance

**Affiliations:** 1 Center for Interdisciplinary Research in Biology, College de France, INSERM U1050, CNRS UMR 7241, Labex Memolife, 75005 Paris, France; 2 University Pierre et Marie Curie, ED 158, Paris Sciences et Lettres, 75005 Paris, France; 3 Laboratory of Neurophysiology, Université Libre de Bruxelles, ULB Neuroscience Institute, Brussels, 1070 Belgium; 4 INRIA, Villeurbanne, 69603 France; 5 University of Lyon, LIRIS UMR 5205, Villeurbanne, 69621 France; 6 WELBIO, Wavre, 1300 Belgium

## Abstract

Dopamine modulates striatal synaptic plasticity, a key substrate for action selection and procedural learning. Thus, characterizing the repertoire of activity-dependent plasticity in striatum and its dependence on dopamine is of crucial importance. We recently unraveled a striatal spike-timing-dependent long-term potentiation (tLTP) mediated by endocannabinoids (eCBs) and induced with few spikes (~5–15). Whether this eCB-tLTP interacts with the dopaminergic system remains to be investigated. Here, we report that eCB-tLTP is impaired in a rodent model of Parkinson’s disease and rescued by L-DOPA. Dopamine controls eCB-tLTP via dopamine type-2 receptors (D_2_R) located presynaptically in cortical terminals. Dopamine–endocannabinoid interactions via D_2_R are required for the emergence of tLTP in response to few coincident pre- and post-synaptic spikes and control eCB-plasticity by modulating the long-term potentiation (LTP)/depression (LTD) thresholds. While usually considered as a depressing synaptic function, our results show that eCBs in the presence of dopamine constitute a versatile system underlying bidirectional plasticity implicated in basal ganglia pathophysiology.

## Abstract (web summary)

Dopamine tightly regulates plasticity at corticostriatal synapses. Here, the authors report that endocannabinoid dependent LTP induced with few spikes in the striatum is impaired in a rodent model of Parkinson’s disease, requires dopamine through presynaptic D2 receptors located on corticostriatal inputs.

## Introduction

Endocannabinoids (eCBs) have emerged as a major signaling system in learning and memory because of their powerful influence on synaptic plasticity, mainly as depressing synaptic function^1,2^. There exists a growing body of evidence that eCBs are also associated with synaptic potentiation^2^. Indeed, eCBs promote long-term potentiation (LTP) at mixed (chemical and electrical) synapses of the goldfish Mauthner cell via intermediary dopaminergic neurons^3^ or at hippocampal CA1 synapses via a GABA_A_ receptor-mediated mechanism^4–6^, facilitation of hippocampal LTP via eCB-induced presynaptic depression of GABAergic transmission^7^, and heterosynaptic short-term potentiation via the astrocytic network^8^. Recently, a direct role of eCBs has been found in promoting LTP in the dentate gyrus^9,10^, the somatosensory cortex^11^, and the dorsolateral striatum^12,13^.

The striatum is a strategic gate extracting pertinent cortical information and a major site of memory formation. Indeed, synaptic plasticity at corticostriatal synapses^14–19^, changes of striatal neuronal activity^14,20^, and corticostriatal coherence^21^ have been associated with the acquisition or extinction in the behavioral repertoire. The striatum receives a dense innervation from midbrain dopaminergic neurons, one of the key players of basal ganglia function in action selection and associative learning^22^. In particular, dopamine plays a crucial role in goal-directed behavior and reinforcement learning, which is dramatically highlighted in neuronal disorders affecting corticostriatal information processing, such as Parkinson’s disease^23^.

Dopamine and synaptic plasticity signaling pathways interaction is required for the induction of the main form of striatal plasticity: the endocannabinoid-mediated long-term depression (eCB-LTD)^24^. At corticostriatal synapses, eCB-LTD has consistently been observed using various cell-conditioning paradigms (high- and low-frequency stimulation, theta-burst protocols, or spike-timing-dependent plasticity)^24–26^. The striatum receives a wide range of patterns of cortical activities from isolated trains of few spikes to prolonged bursting events. While plasticity under prolonged activation is well elucidated, its expression in response to few spikes remains less documented. To this end, we chose spike-timing-dependent plasticity (STDP) as a synaptic Hebbian learning paradigm^27^. Indeed, STDP (tLTP and tLTD) depends on the relative timing between pre- and postsynaptic spikes, and relies on much fewer events (around 100 paired stimulations) than the high- or low-frequency stimulation protocols (hundreds of stimulations). In the striatum, bidirectional STDP with NMDAR-mediated tLTP and eCB-mediated tLTD, has been reported with 100–150 paired stimulations^26,28–32^. Using STDP, we recently reported a new form of plasticity in the dorsolateral striatum: a striatal spike-timing-dependent potentiation (tLTP) induced by few coincident pre- and post-synaptic spikes (~5–15), mediated by eCBs (eCB-tLTP) through a signaling pathway that relies on the activation of type-1 cannabinoid receptor (CB_1_R) and transient receptor potential vanilloid type-1 (TRPV1) and on eCB dynamics^12,13^. Corticostriatal eCB-tLTP relies on postsynaptic synthesis and release of eCBs, which mainly activate presynaptic CB_1_R, and displays a presynaptic locus of plasticity maintenance. We previously showed that the bidirectionality of eCB-dependent STDP in striatum is controlled by eCB-levels: prolonged and moderate release of eCBs leads to eCB-tLTD, whereas brief and large eCB transients produce eCB-tLTP^13^. Moreover, in striatum, eCB-STDP is controlled by the protein kinase A (PKA) and calcineurin activity balance, such as eCB-tLTD requires active calcineurin whereas eCB-tLTP necessitates the activity of presynaptic PKA^12,13^.

Thus, eCBs not only promote depression but also potentiation, i.e., they act as a bidirectional system, depending on the regime of activity pattern on either side of the synapse. The interactions between the dopaminergic system and eCB-LTD have thoroughly been studied and a solid experimental support exists for the efficient modulation of eCB-LTD by dopamine^24^. However, whether and how eCB-tLTP may be modulated by dopamine and consequently be affected in Parkinson’s disease remains unknown. Thus, considering the crucial role of dopamine in striatal physiology and pathophysiology and in particular in synaptic plasticity^22–24,33,34^, we question here the implication of dopamine in the expression of eCB-tLTP. We observe that eCB-tLTP is impaired in a rodent model of Parkinson’s disease and is rescued by L-DOPA treatment. We find that opto-inhibition of dopaminergic neurons prevent eCB-tLTP and that dopamine type-2 receptors (D_2_R) on cortical terminals are required for eCB-tLTP expression. We provide a biologically plausible mathematical model for the dynamics of the implicated signaling pathways. Combining our experimental results and modeling, we show that dopamine controls not only the induction but also the polarity via presynaptic D_2_R (tLTP vs tLTD) of eCB-plasticity by modulating the effective eCB thresholds.

## Results

### Distinct STDP activity patterns induced eCB-tLTP and -tLTD

**Fig. 1:**
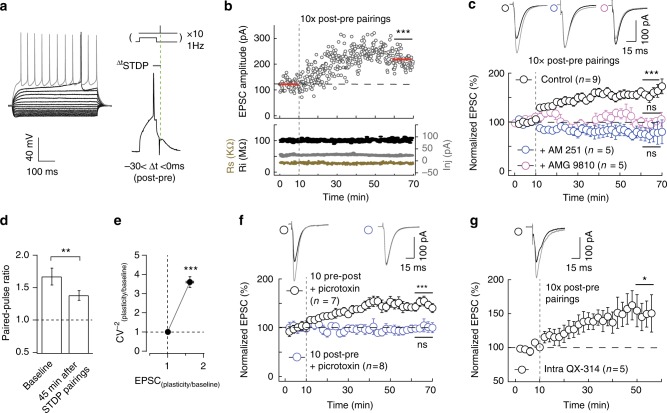
eCB-tLTP is induced by low number of pairings. **a** Characteristic voltage responses of a MSN to a series of 500 ms current pulses from −150 to +180 pA with current steps increasing by 30 pA (black traces) and to +60 pA above spike threshold (grey trace). STDP protocol: a spike-evoked in one MSN was paired with a cortical stimulation repeated 10 times at 1 Hz. ∆*t*_STDP_ indicates the temporal time shift between pre- and postsynaptic stimulations. **b**, **c** 10 post-pre pairings induced tLTP CB_1_R- and TRPV1-activation dependent. **b** Example of tLTP induced by 10 post-pre pairings (∆*t*_STDP_ = −13 ms). Top, EPSC strength before and after 10 pairings (124 ± 4 pA and 203 ± 3 pA, *p* < 0.0001). Bottom, time courses of input resistance (*R*_*i*_) (before, 99 ± 1 MΩ; after, 105 ± 1 MΩ; change of 6%), access resistance (*R*_access_) (before, 30 ± 1 kΩ; after, 29 ± 1 kΩ; change of 3%) and injected current (*I*_inj_) (before, 24 ± 0.3 pA; after, 23 ± 0.2 pA). **c** Summary of STDP experiments showing that eCB-tLTP induced with 10 post-pre pairings (*n* = 9, 9 cells out of 9 resulted in tLTP) is prevented by a specific CB_1_R or TRPV1 inhibitor, AM251 (3 µm, *n* = 5, 1/5 showed tLTP) and AMG9810 (1 µM, *n* = 5, 0/5 showed tLTP), respectively. **d** Summary bar graphs (*n* = 16 MSNs) of paired-pulse cortical stimulations (50 ms interstimulus interval) illustrate a decrease of facilitation after 10 post-pre pairings. This indicates a presynaptic locus of the eCB-tLTP. **e** Mean variance analysis (CV^−2^_plasticity/baseline_, *n* = 21) during baseline and 45 min after STDP pairings indicates a presynaptic locus of the eCB-tLTP. **f** With inhibition of ionotropic GABAergic transmission (picrotoxin 50 μM), tLTP was observed with pre-post (*n* = 7, 6/7 showed tLTP) but not with post-pre (*n* = 8, 1/8 showed tLTP) pairings. GABA controls the time dependence but not the magnitude of eCB-tLTP. **g** Intracellular application of a Na^+^ channel blocker, QX-314 (1 µm, *n* = 5, 5/5 showed tLTP), did not prevent 10 post-pre pairings LTP. Representative traces are the average of 15 EPSCs during baseline (black traces) and 45 min after STDP protocol (grey traces). Vertical grey dashed line indicates the STDP protocol. Error bars represent sem. *p < 0.05; **p < 0.01; ***p < 0.001; ns: not significant by *t*-test, two-tailed (**b**), one sample *t*-test (**c**, **f**, **g**) or Wilcoxon Signed rank test (**c**, **d**)

STDP is a synaptic Hebbian learning rule in which synaptic weight changes depend on the activity on both sides of the synapse^27^. Corticostriatal synapses exhibit a bidirectional eCB-dependent STDP in which eCB-tLTD^12,28–32^ or eCB-tLTP^12,13^ are induced depending on the spike timing (Δ*t*_STDP_) and on the number of pairings (*N*_pairings_). In the dorsolateral striatum, we recently reported that a low number of pairings (*N*_pairings_ = 5–15) induces an eCB-tLTP, dependent on the activation of CB_1_R and TRPV1^12,13^. It is well documented that eCB-LTD (mainly induced with high- or low-frequency stimulation protocols) is controlled by dopamine levels^24^. However, the dopaminergic control of eCB-tLTP remains undocumented. Here, to examine the dopamine dependence of eCB-tLTP, we carried out whole-cell recordings from medium-sized spiny neurons (MSNs) of the dorsolateral striatum (Fig. 1a): after measuring baseline EPSCs for 10 min, recordings were switched to current-clamp to pair a single EPSP induced by presynaptic cortical stimulation with a single postsynaptic spike induced by a brief depolarization of the MSN (Fig. 1a). The STDP protocol consisted in pairing of pre- and postsynaptic stimulations separated by a certain fixed timing interval (Δ*t*_STDP_) and repeated 10 or 100 times at 1 Hz. Δ*t*_STDP_ < 0 when the post-synaptic stimulation occurs before the paired pre-synaptic one (post-pre pairings) (Fig. 1a), whereas Δ*t*_STDP_ > 0 when the pre-synaptic stimulation occurs before the post-synaptic one (pre-post pairings) (Fig. S1a).

We observed that 10 post-pre and 100 pre-post pairings induced a bidirectional anti-Hebbian eCB-STDP with eCB-tLTP induced by 10 post-pre pairings (Fig. 1b, c) and eCB-tLTD by 100 pre-post pairings (Supplementary Fig. 1). GABA operates as a Hebbian/anti-Hebbian switch at corticostriatal synapses^32,35^ because corticostriatal STDP polarity depends on the presence (ex vivo Hebbian STDP^28,29^) or the absence of GABA_A_ receptor antagonists (ex vivo anti-Hebbian STDP^12,13,30,31^, as well as in this study; in vivo anti-Hebbian STDP^36^). We thus recorded STDP in the absence of GABA_A_R antagonist to preserve the local striatal microcircuits and the anti-Hebbian polarity as observed in vivo^36^. Figure 1b and Supplementary Figure 1b show examples of tLTP and tLTD induced by 10 post-pre and 100 pre-post pairings, respectively. To summarize, 10 post-pre STDP pairings (−30 < Δ*t*_STDP_ < 0 ms) induced tLTP (mean value of the EPSC amplitude recorded 50 min after STDP protocol: 158 ± 11%, *p* = 0.0006, *n* = 9), which was prevented by AM251 (3 μM), a CB_1_R specific inhibitor (80 ± 14%, *p* = 0.2223, *n* = 5) or with AMG9810 (1 μM), a TRPV1-specific inhibitor (98 ± 7%, *p* = 0.7723, *n* = 5) (Fig. 1c) as recently reported^12,13^. 100 pre-post pairings (0 < Δ*t*_STDP_ < +30 ms) induced tLTD (71 ± 10%, *p* = 0.0253, *n* = 7), which was prevented by AM251 (103 ± 7%, *p* = 0.7500, *n* = 7) or with AMG9810 (99 ± 10%, *p* = 0.9197, *n* = 5) (Supplementary Fig. 1c–d) confirming previous reports^13,28–32^. Thus, we confirm that eCB system encodes for bidirectional striatal plasticity depending on the number and order of pairings.

CB_1_R being located at the presynaptic terminals of the corticostriatal pathway^1,24^, the locus of eCB-tLTP maintenance would likely be presynaptic. We applied presynaptic paired-pulses (50 ms interpulse interval) before and 45 min after STDP pairings, and observed a EPSC paired-pulse facilitation^12,13^. We observed a decrease of the paired-pulse ratio (paired-pulse ratio_plasticity/baseline_ = 0.855 ± 0.04, *p* = 0.0032, *n* = 16) 45 min after the 10 post-pre pairings (Fig. 1d), which indicates a presynaptic locus of eCB-tLTP. The mean variance analysis of EPSCs gave a CV^−2^ value of 3.55 ± 0.60 (*p* < 0.0001, *n* = 21), which also indicates a presynaptic locus for eCB-tLTP (Fig. 1e).

We investigated the impact of GABAergic networks on eCB-tLTP, which could arise from a decrease of GABA release, as reported for hippocampal eCB-LTP^4,7^. When ionotropic GABAergic transmission was blocked with picrotoxin (50 μM), tLTP was observed for 10 pre-post pairings (142 ± 18%, *p* = 0.0008, *n* = 7), whereas no detectable plasticity was found for 10 post-pre pairings (99 ± 19%, *p* = 0.8909, *n* = 8) (Fig. 1f). tLTP magnitudes observed in control and picrotoxin conditions were not significantly different (*p* = 0.4575). Therefore, GABAergic circuits do not control the expression of eCB-tLTP but control its timing dependence, in agreement with the GABA effects reported for 100 pairings NMDA-tLTP and eCB-LTD^32,35^.

The subthreshold postsynaptic depolarization itself is a key factor in the induction of striatal plasticity^37^. We tested whether postsynaptic spikes were required for eCB-tLTP induction. To this aim, we delivered intracellularly in the postsynaptic recorded MSN via the patch pipette the QX-314 (10 μM), a Na^+^ channel inhibitor, and observed tLTP with 10 post-pre pairings (158 ± 21%, *p* = 0.0483, *n* = 5; Fig. 1f). Therefore, subthreshold depolarizations (30 ms duration) appeared sufficient to induce striatal eCB-tLTP.

### eCB-tLTP is impaired in Parkinson’s and rescued by L-DOPA

**Fig. 2:**
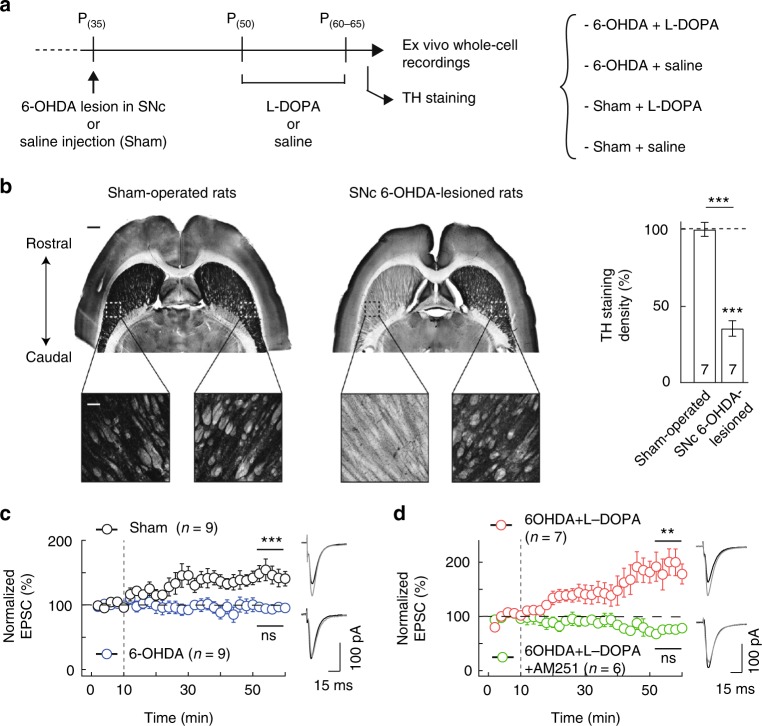
eCB-tLTP is impaired in a rat model of Parkinson’s and rescued by L-DOPA. **a** Protocols of the 6-OHDA lesion (or sham) in P_35_ rats followed 2 weeks after by chronic L-DOPA treatment (or saline) for 10 days. **b** Unilateral 6-OHDA injection in SNc led to degeneration of dopaminergic nigral neurons and a loss of their striatal afferences as illustrating by TH immunostaining in horizontal brain slices. Note that sham-operated rats display equivalent TH staining in both striata. Scale bars: 1 mm and 200 µm. Right panel: summary bar graph of TH staining quantification. **c** 10 post-pre pairings induced eCB-tLTP in sham-operated rats (*n* = 9, 9/9 cells showed tLTP) while no plasticity was observed in 6-OHDA-lesioned rats (*n* = 9, 1/9 cells showed tLTP). **d** Chronic L-DOPA treatment consisting in twice daily injection of L-DOPA (10 mg/kg) for 10 days, 2 weeks after 6-OHDA lesion allowed to recover tLTP (*n* = 7, 6/7 cells showed tLTP) induced with 10 post-pre pairings. This tLTP was CB_1_R-mediated since prevented by AM251 (3 µm, *n* = 6, 0/6 cells showed tLTP) in 6-OHDA-lesioned rats treated with L-DOPA. Representative traces are the average of 15 EPSCs during baseline (black traces) and 45 min after STDP protocol (grey traces). Vertical grey dashed line indicates the STDP protocol. Error bars represent sem. **p < 0.01; ***p < 0.001; ns: not significant by one sample *t*-test (**c**, **d**) or Wilcoxon Signed rank test (**b**)

Whereas NMDAR-LTP and eCB-LTD striatal synaptic plasticity are dependent on dopamine and therefore are impacted in Parkinson’s disease^23,24,34^, it is not known whether eCB-tLTP is affected in Parkinson’s disease. To address this question, we used a rat model of Parkinson’s disease in which dopaminergic transmission is impaired. We performed unilateral lesion of the substantia nigra *pars compacta* (SNc) with 6-hydroxy-dopamine (6-OHDA), a neurotoxic synthetic organic compound which leads, when associated with desipramine, to the selective degeneration of dopaminergic neurons (Fig. 2a, b). We observed a massive degeneration of nigral dopaminergic neurons and of their striatal terminals as shown by the dramatic decrease of striatal tyrosine hydroxylase (TH) staining by 64 ± 5% (*p* < 0.0001, *n* = 7) 2 weeks after the 6-OHDA lesions at P_50_ (Fig. 2b). We also used sham-operated animals with saline injection for comparison with the 6-OHDA-lesioned rats (Fig. 2a, b). We first verified that tLTP was induced with 10 post-pre pairings in adult control rats (i.e., without any surgery and recorded at similar age than the sham-operated and 6-OHDA-lesioned rats, P_(60–65)_) (164 ± 16%, *p* = 0.0034, *n* = 10; Supplementary Fig. 2a) and in sham-operated rats (147 ± 7%, *p* = 0.0002, *n* = 9; Fig. 2c). The tLTP induced in adult control animals and in sham-operated rats were not different (*p* = 0.7019). In contrast, in the 6-OHDA-lesioned animals, 10 post-pre pairings failed to induce any plasticity (94 ± 5%, *p* = 0.2741, *n* = 9; Fig. 2c) thus showing that the degeneration of dopaminergic neurons is deleterious for the induction of eCB-tLTP.

We next tested whether treatment with L-3,4-dihydroxyphenylalanine (L-DOPA; Fig. 2a), a mainstay for symptomatic treatment of Parkinson’s disease, could rescue eCB-tLTP in 6-OHDA-lesioned rats. In slices obtained from 6-OHDA-lesioned animals treated with L-DOPA, 10 post-pre pairings caused tLTP (171 ± 19%, *p* = 0.0096, *n* = 7; Fig. 2d); this tLTP was not different from the tLTP obtained in sham-operated rats (*p* = 0.3481). tLTP was also observed in the sham-operated animals L-DOPA-treated (164 ± 20%, *p* = 0.0201, *n* = 7; Supplementary Fig. 2b) and this tLTP was not different from the one obtained in sham-operated rats (*p* = 0.4038) or in 6-OHDA-lesioned animals treated with L-DOPA (*p* = 0.7791). In 6-OHDA-lesioned animals L-DOPA-treated, tLTP induced with 10 post-pre pairings was CB_1_R-mediated since prevented by AM251 (81 ± 6%, *p* = 0.0244, *n* = 6; Fig. 2d). Therefore, eCB-tLTP was impaired in a rodent model of Parkinson’s disease and was rescued by L-DOPA treatment.

### Dopamine is required for eCB-tLTP induction

**Fig. 3:**
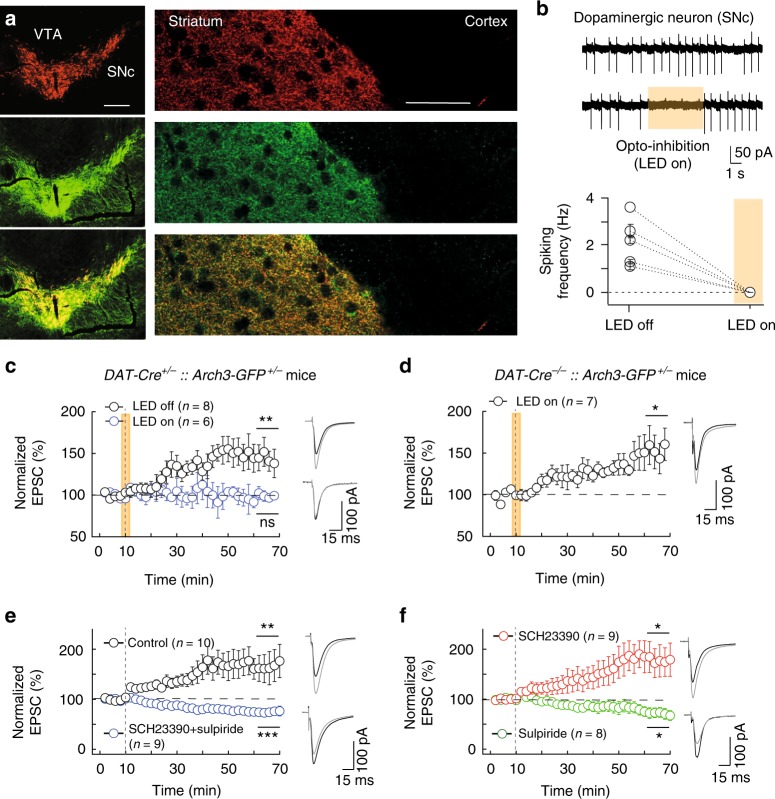
eCB-LTP requires dopamine release during pairings and is D_2_R-dependent. **a**–**d** Induction of eCB-LTP requires dopamine release during the STDP paired-activity paradigm. **a** Double immunostaining for tyrosine hydroxylase (indicating dopaminergic cell bodies in SNc and dopaminergic afferences in striatum; upper panels) and GFP (indicating Arch3-expression; middle panels) in SNc (left panels) and in striatum (right panels); merge images are shown in the lower panels. Scale bars: 500 µm (SNc) and 50 µm (striatum). **b** Spontaneous firing of dopaminergic neurons recorded in cell-attached mode in *DAT-Cre*^+/−^*::Arch3-GFP*^+/−^ mice with and without light (upper traces). Summary graph showing all experiments performed in *DAT-Cre*^+/−^*::Arch3-GFP*^+/−^ mice with and without opto-inhibition. **c** 15 post-pre pairings induced tLTP (*n* = 8, 6/8 cells showed tLTP) in *DAT-Cre*^+/−^*::Arch3-GFP*^+/−^ mice without opto-stimulation (LED off), while opto-stimulation (LED on) during STDP pairings prevents tLTP induction (*n* = 6, 1/6 cells showed tLTP). This illustrates that eCB-tLTP requires DA release during STDP pairings. **d** 15 post-pre pairings induced tLTP with concomitant opto-stimulation (16 s duration) during STDP protocol in *DAT-Cre*^*−/−*^*::Arch3*^*+/+*^ mice (*n* = 7, 6/7 cells showed tLTP). In panels (**b**–**d**), the yellow areas illustrate when LED was on to promote an opto-inhibition. **e**, **f** 10 post-pre pairings (−30 < ∆*t*_STDP_ < 0 ms) induced tLTP is D_2_R-activation dependent. Summary of STDP experiments showing that eCB-tLTP induced with 10 post-pre pairings (*n* = 10) is prevented by the co-application of antagonists of D_1_R and D_2_R, SCH23390 (4 µM) and sulpiride (10 µM) (*n* = 9, 8/9 cells showed tLTD and 1/9 showed no plasticity) (**e**), and with the D_2_R antagonist, sulpiride (10 µM, *n* = 8, 0/8 cells showed tLTP and 5/8 showed tLTD) but tLTP was left unaffected by the sole application of the D_1_R antagonist, SCH23390 (4 µM, *n* = 9, 8/9 cells showed tLTP) (**f**). Representative traces are the average of 15 EPSCs during baseline (black traces) and 45 min after STDP protocol (grey traces). Vertical grey dashed line indicates the STDP protocol. Error bars represent sem. *p < 0.05; **p < 0.01; ***p < 0.001; ns: not significant by one sample *t*-test (**c**–**f**)

Cortical stimulation enhances dopamine release in the dorsal striatum^38–40^. We investigated if dopamine was required during the few pairings of the induction phase of eCB-tLTP. To this intent, we opto-inhibited dopaminergic neurons during the few pairings responsible for eCB-tLTP in *DAT-Cre*^+/−^*::Arch3-GFP*^+/−^ mice. We confirmed that Arch3-GFP expression was restricted to dopaminergic neurons and terminals in the striatum with TH and GFP immunostainings (*n* = 10) in SNc and dorsal striatum (Fig. 3a). We ensured the efficiency of the opto-inhibition by cell-attached recordings of the spontaneous spiking activity of dopaminergic neurons in the SNc from *DAT-Cre*^*+/−*^*::Arch3-GFP*^*+/−*^ mice. Upon photostimulation, we observed an absence of spikes (2.1 ± 0.5 Hz without light and 0 Hz with light, *n* = 5; with *I*_inj_ = 0 pA) (Fig. 3b) and a hyperpolarization of the resting membrane potential (−19 ± 5 mV, *n* = 5; with *I*_inj_ = 100 pA). We first verified that few pairings successfully induced tLTP (without photostimulation) in *DAT-Cre*^+/−^*::Arch3-GFP*^+/−^ mice; we observed tLTP with 15 post-pre pairings (143 ± 12%, *p* = 0.0091, *n* = 8; Fig. 3c). 5–10 post-pre pairings (with a single postsynaptic spike) were sufficient to induce potent tLTP in rat whereas in C57BL/6 mice 15 pairings (with 2–3 postsynaptic spikes) were necessary to trigger tLTP. In *DAT-Cre*^+/−^*::Arch3-GFP*^+/−^ mice, photostimulation concomitantly with the STDP protocol prevented tLTP (102 ± 8%, *p* = 0.7943, *n* = 6; Fig. 3c). Here, the 16s photostimulation of Arch3 was far below artifactual local transmitter release reported for long (5 min) Arch3 activation^41^. As a control we ensured that photostimulation itself in *DAT-Cre*^*−/−*^*::Arch3-GFP*^+/−^ mice did not impair tLTP induction: tLTP was induced with 15 post-pre pairings applied concomitantly with photostimulation (140 ± 12%, *p* = 0.0151, *n* = 7; Fig. 3d). Therefore, dopamine release during the STDP pairings is necessary for the induction of eCB-tLTP.

### eCB-tLTP and -tLTD depend on distinct dopamine receptor

In the striatum, the principal subtypes of dopamine receptors are D_1_ and D_2_ receptors^33^. These receptors have opposite effects: D_1_R activates adenylyl cyclase and PKA via G_olf_-coupled receptor signaling, and D_2_R inhibits them via G_i/o_-coupled receptor. We questioned which dopaminergic receptor subtype was involved in eCB-tLTP induced by 10 post-pre pairings. Bath-applied SCH23390 (4 µM, a D_1_R antagonist) and sulpiride (10 µM, a D_2_R antagonist) prevented eCB-tLTP. Indeed, we even observed a depression instead of tLTP (77 ± 4%, *p* = 0.0002, *n* = 9; Fig. 3e). We next selectively inhibited either D_1_R or D_2_R. When we inhibited D_1_R (SCH23390), we observed tLTP (169 ± 21%, *p* = 0.0113, *n* = 9) while no tLTP could be elicited with sulpiride (73 ± 8%, *p* = 0.0117, *n* = 8) (Fig. 3f). Hence, we observed that the prolonged inhibition of D_2_R (as well as both D_1_R and D_2_R) flipped tLTP into tLTD. In an attempt to determine whether D_2_R and CB_1_R share a common, parallel, or cooperative pathway^42^, we next bath-applied quinpirole (10 µM), a D_2_R agonist, concomitantly with 10 post-pre pairings, and observed tLTP (146 ± 16%, *p* = 0.0334, *n* = 6; Supplementary Fig. 3), which was not different from the control tLTP (*p* = 0.1871). This observation is not in favor of cooperative or common pathways hypothesis for D_2_R and CB_1_R, but points more likely toward parallel pathways^42^.

Secondly, we compared the dependence on dopamine receptor subtypes of eCB-tLTP with eCB-tLTD. We observed that the eCB-tLTD induced by 100 pre-post pairings was impaired by SCH23390 and sulpiride, (106 ± 14%, *p* = 0.6714, *n* = 6; Supplementary Fig. 4a). We next selectively inhibited either D_1_R or D_2_R subtypes. The plasticity induced by 100 pre-post pairings was lost (105 ± 16%, *p* = 0.7405, *n* = 8) when we inhibited D_1_R with SCH23390, whereas we observed tLTP, instead of tLTD, with sulpiride (199 ± 36%, *p* = 0.0335, *n* = 7; Supplementary Fig. 4b).

Altogether, these experiments indicate that eCB-tLTP is D_2_R-mediated but does not depend on D_1_R whereas eCB-tLTD is mediated by both D_1_R and D_2_R.

### What is the location of the D_2_R required for eCB-tLTP?

**Fig. 4:**
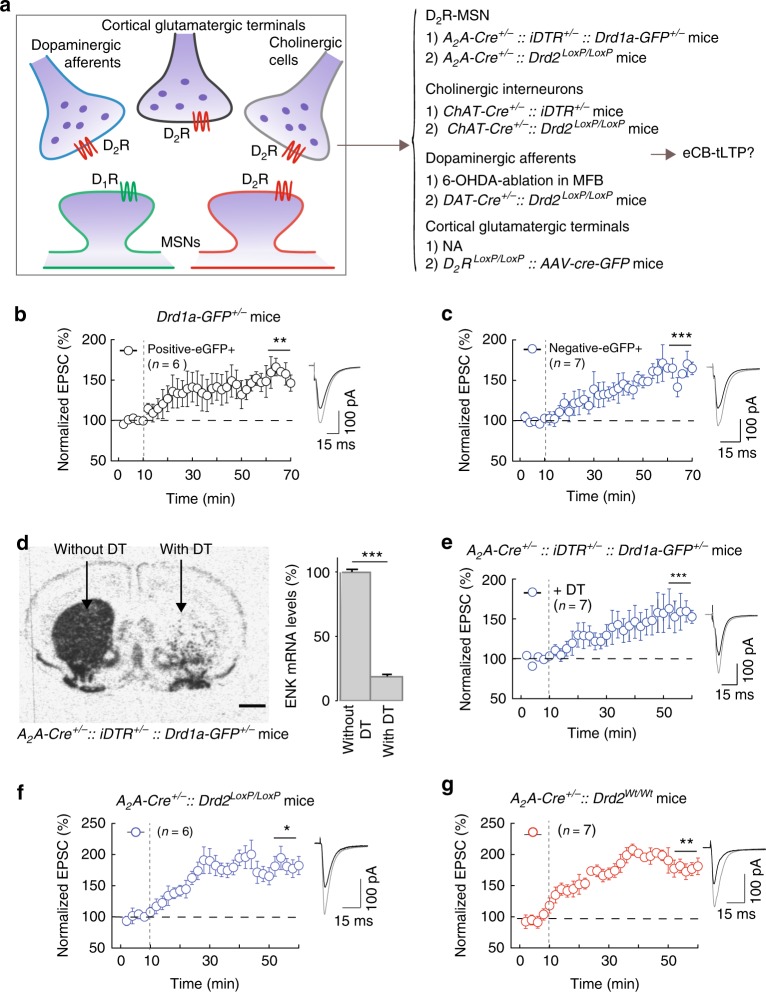
eCB-tLTP does not depend on D_2_R expressed by D_2_R-MSNs. **a** Location of D_2_R in striatum expressed in striato-pallidal MSNs, cholinergic interneurons, SNc dopaminergic and cortical glutamatergic afferents. Right: the two complementary strategies to identify the D_2_R involved in eCB-tLTP: (1) genetic-ablation of D_2_R-MSN or cholinergic interneurons (using DTR and stereotaxic DT injection) and 6-ODHA-ablation of dopaminergic cells in the MFB, and (2) D_2_R-cKO in D_2_R-MSN, cholinergic interneurons, dopaminergic cells, or corticostriatal glutamatergic afferents; NA accounts for the impossibility to use a genetic-ablation of corticostriatal afferents without losing the studied EPSCs. **b**, **c** tLTP induced with 15 post-pre pairings is observed in both striato-nigral (D_1_R-eGFP positive neurons, D_1_R-eGFP^+^, *n* = 6, 5/6 cells showed tLTP) (**b**) and striato-pallidal (D_1_R-eGFP negative neurons, non-D_1_R-eGFP^+^, *n* = 7, 7/7 cells showed tLTP) (**c**) MSNs. **d**, **e** Experiments of plasticity expression in genetically-ablated mice for A_2_A-expressing neurons in the dorsal striatum. **d** In situ hybridization autoradiograms (left) and quantification (right bar graph, *n* = 5 mice) of enkephalin mRNA in striatum caudal level of coronal sections in *A*_*2A*_*-Cre*^+/−^*::iDTR*^+/−^*::Drd1a-GFP*^+/−^ mice injected with DT stereotaxically in the dorsal striatum (right hemisphere) and without DT injection (left hemisphere). Scale bars: 1000 µm. **e** In mice in which D_2_R-MSNs were ablated (*A*_*2A*_*-Cre*^+/−^*::iDTR*^+/−^*::Drd1a-GFP*^+/−^ mice injected with DT stereotaxically in the dorsal striatum) tLTP was induced with 15 post-pre pairings (*n* = 7, 7/7 cells showed tLTP). **f**, **g** Experiments of plasticity expression in selective cKO for D_2_R expressed by striatal D_2_R-MSNs. **f** In mice in which D_2_R was specifically knocked-out in D_2_R-MSNs (*A*_*2A*_*-Cre*^+/−^*::Drd2*^*LoxP/LoxP*^ mice) tLTP was induced with 15 post-pre pairings (*n* = 6, 5/6 cells showed tLTP). **g** tLTP was observed in mice serving as the Cre driver control (*A*_*2A*_*-Cre*^+/−^*::Drd2*^*Wt/Wt*^ mice, *n* = 7, 6/7 cells showed tLTP). Representative traces are the average of 15 EPSCs during baseline (black traces) and 45 min after STDP protocol (grey traces). Vertical grey dashed line indicates the STDP protocol. Error bars represent sem. *p < 0.05; **p < 0.01; ***p < 0.001 by one sample *t*-test (**b**, **c**–**g**) or Wilcoxon Signed rank test (**b**)

We next questioned the location of the D_2_R involved in the induction of eCB-tLTP. D_2_R are expressed at different locations in the dorsal striatum: postsynaptically in D_2_R-expressing MSNs^43^ and presynaptically in cholinergic interneurons^44^, nigrostriatal dopaminergic neurons^45^, and glutamatergic cortical afferents^46,47^ (Fig. 4a). To identify the D_2_R involved in the eCB-tLTP, we opted for two complementary strategies: (1) we genetically-ablated selectively D_2_R-MSN or cholinergic interneurons (using Cre-mediated expression of the diphtheria toxin receptor (DTR) and stereotaxic diphtheria toxin (DT) injection^48^) and 6-ODHA-ablated selectively dopaminergic cells in the medial forebrain bundle (MFB), and (2) we selectively knocked-out the D_2_R expressed at D_2_R-MSN, cholinergic interneurons, dopaminergic cells, or corticostriatal glutamatergic terminals, and next examined whether eCB-tLTP could still be observed (Fig. 4a). Note that it was not possible to use the first strategy (i.e., genetic ablation) for corticostriatal afferents without impairing the corticostriatal transmission.

### D_2_R on D_2_R-MSNs are not required for eCB-tLTP

We first questioned the postsynaptic localization of the D_2_R involved in eCB-tLTP at the level of the MSNs (Fig. 4a). Due to the segregation of expression of D_1_R and D_2_R among MSNs in mice (D_1_R-like and D_2_R-like for the direct and indirect pathways, respectively)^43^, roughly half of the MSNs are expected to be D_2_R-expressing neurons. If eCB-tLTP was supported by the postsynaptic D_2_R MSNs, one would expect to induce eCB-tLTP in ~50% of the (randomly chosen) recorded MSNs. eCB-tLTP was successfully induced in 83% (*n* = 27) of the (randomly chosen) recorded MSNs, which does not favor the hypothesis of involvement of D_2_R expressed by MSNs in eCB-tLTP. To confirm this, we used transgenic D_1_R-eGFP mice to induce eCB-tLTP specifically in D_1_R-eGFP^+^ or non-D_1_R-eGFP^+^ MSNs (Fig. 4b, c). We observed tLTP in both D_1_R-eGFP^+^ (148 ± 8%, *p* = 0.0033, *n* = 6; Fig. 4b) and non-D_1_R-eGFP^+^ (150 ± 7%, *p* = 0.0006, *n* = 7; Fig. 4c) MSNs, indicating that tLTP can be induced in both striatopallidal and striatonigral MSNs and that a postsynaptic D_2_R in MSNs is a priori less likely to be involved in eCB-tLTP.

To further test the involvement of the D_2_R-MSNs, we selectively ablated these neurons by Cre-mediated expression of DTR and DT injection^48^. To inactivate D_2_R in the specific neuronal population in the striatum of adult mice we use genetic ablation to avoid developmental effect or compensation. Moreover, it has been shown that the ablation of 40–45% of the striatal neurons in adult mice has no noticeable effect on others neuronal populations^48^. We placed Cre recombinase under the control of A_2A_R promoter because in the striatum A_2A_R expression is restricted to D_2_R-MSNs. The DT was stereotaxically injected into the dorsal striatum to produce ablation of D_2_R-MSNs. In those *A*_*2A*_*-Cre*^+/−^*::iDTR*^+/−^*::Drd1a-GFP*^+/−^ transgenic mice after stereotaxic injection of DT, we found no detectable D_2_R^+^ cells but only D_1_R-GFP MSNs, whereas in the contra-lateral hemisphere where DT had not been injected both populations of D_1_R^+^- and D_2_R^+^-MSNs were observed (Fig. 4d). Whole-cell recordings of D_1_R-GFP MSNs, from *A*_*2A*_*-Cre*^+/−^*::iDTR*^+/−^*::Drd1a-GFP*^+/−^ mice injected bilaterally with DT, showed that 15 post-pre pairings were still able to cause tLTP (146 ± 7%, *p* = 0.0007, *n* = 7; Fig. 4e). As a control, we similarly injected DT in mice lacking DTR expression (*A*_*2A*_*-Cre*^*−/−*^*::iDTR*^+/−^*::Drd1a-GFP*^+/−^) and also observed tLTP (137 ± 10%, *p* = 0.0174, *n* = 6; Supplementary Fig. 5a) that was not different from tLTP obtained with ablation of D_2_R-MSN (*p* = 0.3566).

We next generated selective conditional knock-out (cKO) mice for D_2_R expressed by D_2_R-MSN (*A*_*2A*_*-Cre*^+/−^*::Drd2*^*LoxP/LoxP*^ mice). We observed expression of tLTP induced by 15 post-pre pairings in MSNs from *A*_*2A*_*-Cre*^+/−^*::Drd2*^*LoxP/LoxP*^ mice (181 ± 24%, *p* = 0.0192, *n* = 6; Fig. 4f). As the Cre driver control, we used the *A*_*2A*_*-Cre*^+/−^*::Drd2*^*Wt/Wt*^ mice and observed tLTP following 15 post-pre pairings (172 ± 18%, *p* = 0.0070, *n* = 7; Fig. 4g), which was not different from tLTP observed in *A*_*2A*_*-Cre*^+/−^*::Drd2*^*LoxP/LoxP*^ mice (*p* > 0.9999).

These results show that postsynaptic D_2_R located on D_2_R-MSNs are not required for eCB-tLTP but thus should rely on the activation of presynaptically located D_2_R.

### D_2_R on cholinergic interneurons are not required for eCB-tLTP

**Fig. 5:**
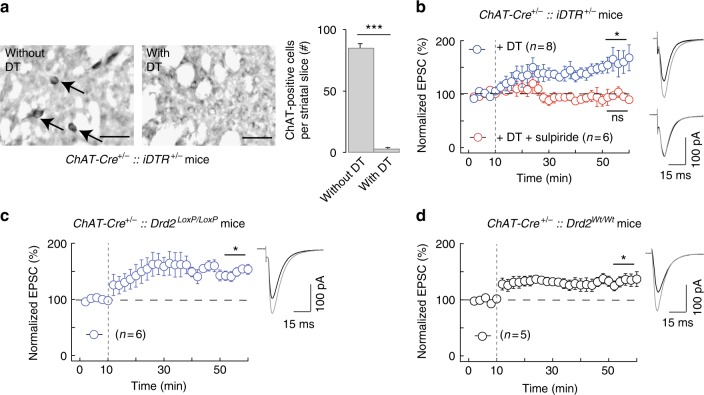
eCB-tLTP does not depend on D_2_R expressed by cholinergic interneurons. **a**, **b** Experiments of plasticity expression in genetically-ablated mice for ChAT-expressing neurons in the dorsal striatum. **a** ChAT immunostaining in *ChAT-Cre*^+/−^*::iDTR*^+/−^ (left panels) mice injected with DT stereotaxically in the dorsal striatum (right hemisphere) and without DT injection (left hemisphere); quantification (right bar graph) of the number of Ach-positive cells per striatal slices (*n* = 6 mice). **b** tLTP was induced with 15 post-pre pairings in *ChAT-Cre*^+/−^*::iDTR*^+/−^ mice (*n* = 8, 8/8 cells showed tLTP) injected bilaterally with diphtheria toxin in the dorsal striatum. tLTP observed in cholinergic interneuron-ablated mice was D_2_R-mediated because it was prevented by sulpiride (10 µM, *n* = 6, 2/6 cells showed tLTP). **c**, **d** STDP expression in selective cKO for D_2_R expressed by cholinergic interneurons. **c** In mice in which D_2_R was specifically knocked-out in cholinergic interneurons (*ChAT-Cre*^+/−^*::Drd2*^*LoxP/LoxP*^ mice) tLTP was induced with 15 post-pre pairings (*n* = 6, 6/6 cells showed tLTP). **d** tLTP was observed in mice serving as the Cre driver control (*ChAT-Cre*^+/−^*::Drd2*^*Wt/Wt*^ mice, *n* = 5; 4/5 cells showed tLTP). Representative traces are the average of 15 EPSCs during baseline (black traces) and 45 min after STDP protocol (grey traces). Vertical grey dashed line indicates the STDP protocol. Error bars represent sem. *p < 0.05; ***p < 0.001; ns: not significant by Wilcoxon Signed rank test (**a**) or one sample *t*-test (**b**–**d**)

Next, we first selectively ablated striatal cholinergic interneurons by Cre-mediated expression of DTR and DT injection to test the participation of the D_2_R express by striatal cholinergic interneurons in eCB-tLTP (Fig. 5a). Cre recombinase was under the control of ChAT promoter because in the striatum ChAT expression is restricted to cholinergic interneurons. Ablation of cholinergic interneurons was induced by DT, which was stereotaxically injected into the dorsal striatum. We verified that in *ChAT-Cre*^+/−^*::iDTR*^+/−^ mice, there was no detectable ChAT cells after stereotaxic injection of DT whereas in the contra-lateral hemisphere (without DT injection) a positive ChAT staining was observed (Fig. 5a). In *ChAT-Cre*^+/−^*::iDTR*^+/−^ mice injected bilaterally with DT, 15 post-pre pairings induced tLTP (161 ± 20%, *p* = 0.0182, *n* = 8; Fig. 5b). We verified that this tLTP was D_2_R-mediated by applying sulpiride (10 µM) and no plasticity was observed (99 ± 10%, *p* = 0.9160, *n* = 6; Fig. 5b). As a further control, we similarly injected DT in mice lacking DTR expression (*ChAT-Cre*^*−/−*^*::iDTR*^+/−^) and observed a tLTP (150 ± 13%, *p* = 0.0171, *n* = 5; Supplementary Fig. 5b) that was similar to tLTP obtained with ablation of cholinergic interneurons (*p* = 0.8221).

In the second step, we generated selective cKO mice for D_2_R expressed in cholinergic interneurons (*ChAT-Cre*^+/−^*::Drd2*^*LoxP/LoxP*^ mice). In *ChAT-Cre*^+/−^*::Drd2*^*LoxP/LoxP*^ mice, 15 post-pre pairings induced tLTP in MSNs (169 ± 18%, *p* = 0.0111, *n* = 6; Fig. 5c). As the Cre driver control, we used the *ChAT-Cre*^+/−^*::Drd2*^*Wt/Wt*^ mice and observed tLTP following 15 post-pre pairings (135 ± 9%, *p* = 0.0175, *n* = 5; Fig. 5d), which was not different from tLTP observed in *ChAT-Cre*^+/−^*::Drd2*^*LoxP/LoxP*^ mice (*p* = 0.0823).

These results indicate that eCB-tLTP does not depend on D_2_R-activation located in cholinergic interneurons.

### D_2_R on dopaminergic neurons are not required for eCB-tLTP

**Fig. 6:**
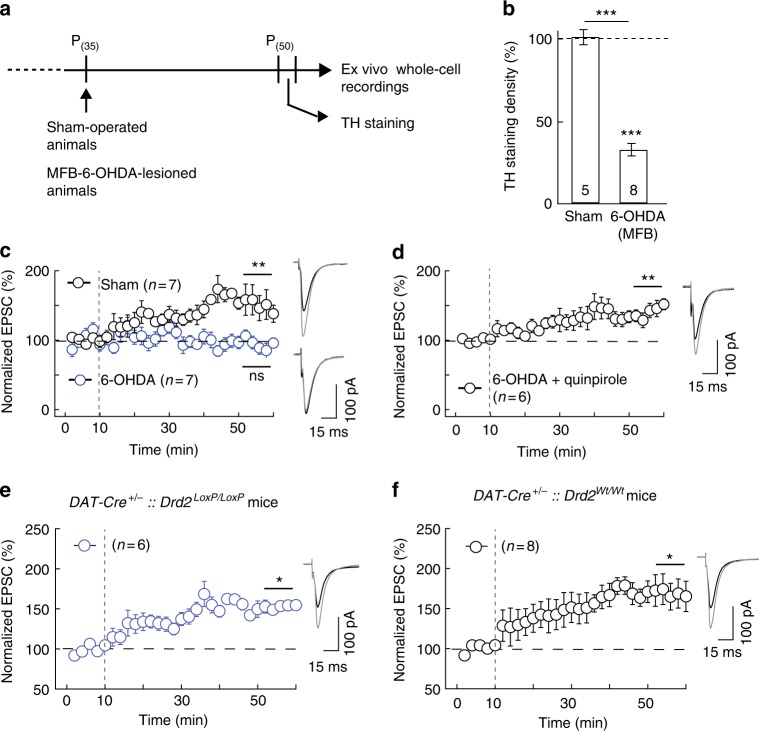
eCB-tLTP does not depend on D_2_R expressed by dopaminergic neurons. **a**–**d** Experiments of plasticity expression in 6-ODHA-ablated dopaminergic cells in the MFB. **a** Scheme of the protocol showing the time course of the in vivo 6-OHDA lesions and ex vivo electrophysiological recordings. **b** Unilateral 6-OHDA injection in MFB led to degeneration of dopaminergic nigral neurons, illustrated by a loss of their striatal afferences as illustrating by the summary bar graph of TH immunostaining quantification. Sham-operated rats display equivalent TH staining in both striata. **c** 10 post-pre pairings induced tLTP in sham-operated (*n* = 7, 7/7 cells showed tLTP) rats whereas tLTP was impaired in 6-OHDA-lesioned rats (*n* = 7, 1/7 cells showed tLTP), and **d** rescued with a D_2_R agonist, quinpirole (10 µM, *n* = 6, 5/6 cells showed tLTP). **e**, **f** STDP expression in selective cKO for D2R expressed by dopaminergic neurons. **e** In mice in which D_2_R was specifically knocked-out in dopaminergic neurons (*DAT-Cre*^+/−^*::Drd2*^*LoxP/LoxP*^ mice) tLTP was induced with 15 post-pre pairings (*n* = 6, 6/6 cells showed tLTP). **f** tLTP was observed in mice serving as the Cre driver control (*DAT-Cre*^+/−^*::Drd2*^*Wt/Wt*^ mice, *n* = 8; 6/8 cells showed tLTP). Representative traces are the average of 15 EPSCs during baseline (black traces) and 45 min after STDP protocol (grey traces). Vertical grey dashed line indicates the STDP protocol. Error bars represent sem. *p < 0.05; **p < 0.01; ***p < 0.001; ns: not significant by Wilcoxon Signed rank test (**b**) or one sample *t*-test (**c**–**f**)

To evaluate the potential role of presynaptic D_2_R in the dopaminergic neurons, we first lesioned dopaminergic neurons with 6-OHDA. We performed in vivo stereotaxic 6-OHDA injection (with desipramine) within the MFB of P_35_ rats (Fig. 6a) and striatal TH staining was decreased by 77 ± 3% (*p* < 0.0001, *n* = 8) 2 weeks after lesion at P_50_ (Fig. 6b). In parallel, we used control (P_50_ rats without any surgery) and sham-operated (P_35_ rats with saline injection instead of 6-OHDA and recorded at P_50_) animals (Fig. 6c and Supplementary Fig. 5c). We first verified that tLTP was induced with 10 post-pre pairings in P_50_ control rats (158 ± 16%, *p* = 0.0168, *n* = 6; Supplementary Fig. 5c) and sham-operated rats (147 ± 10%, *p* = 0.0032, *n* = 7; Fig. 6c); both tLTP show similar magnitude (*p* = 0.7133). As expected, in 6-OHDA animals we did not observe any plasticity with 10 post-pre pairings (90 ± 7%, *p* = 0.1916, *n* = 7; Fig. 6c). We next bath-applied quinpirole (10 µM), a selective agonist of D_2_R, to overcome the lack of dopamine, and tLTP was observed (141 ± 9%, *p* = 0.0076, *n* = 6; Fig. 6d); this tLTP was not different from those observed in sham-operated rats (*p* = 0.7984). In conclusion, tLTP could be observed (with quinpirole) in 6-OHDA-lesioned animals.

In the second step, we generated selective cKO mice for D_2_R expressed in dopaminergic cells (*DAT-Cre*^+/−^*::Drd2*^*LoxP/LoxP*^ mice). Whole-cell recordings of MSNs from *DAT-Cre*^+/−^*::Drd2*^*LoxP/LoxP*^ mice showed that 15 post-pre pairings induced tLTP (154 ± 15%, *p* = 0.0166, *n* = 6; Fig. 6e). As the Cre driver control, we used the *DAT-Cre*^+/−^*::Drd2*^*Wt/Wt*^ mice and observed tLTP following 15 post-pre pairings (169 ± 22%, *p* = 0.0157, *n* = 8; Fig. 6f), which was not different from tLTP observed in *DAT-Cre*^+/−^*::Drd2*^*LoxP/LoxP*^ mice (*p* = 0.7193).

Altogether, these results show that presynaptic D_2_R located on nigrostriatal dopaminergic afferents are not required for eCB-tLTP.

We recorded MSNs from *Cre*^*−/−*^*::Drd2*^*LoxP/LoxP*^ mice as the floxed gene control for the selective D_2_R-cKO mice experiments. 15 post-pre pairings induced tLTP in *Cre*^*−/−*^*::Drd2*^*LoxP/LoxP*^ mice (156 ± 12%, *p* = 0.0052, *n* = 7; Supplementary Fig. 5d). We did not observe any difference between tLTP recorded in MSNs in the floxed gene control (*Cre*^*−/−*^*::Drd2*^*LoxP/LoxP*^ mice) and the Cre driver controls (*A*_*2A*_*-Cre*^+/−^*::Drd2*^*Wt/Wt*^, *ChAT-Cre*^+/−^*::Drd2*^*Wt/Wt*^, and *DAT-Cre*^+/−^*::Drd2*^*Wt/Wt*^ mice) (ANOVA, *p* = 0.5524).

### eCB-tLTP depends on D_2_R in corticostriatal pyramidal cells

**Fig. 7:**
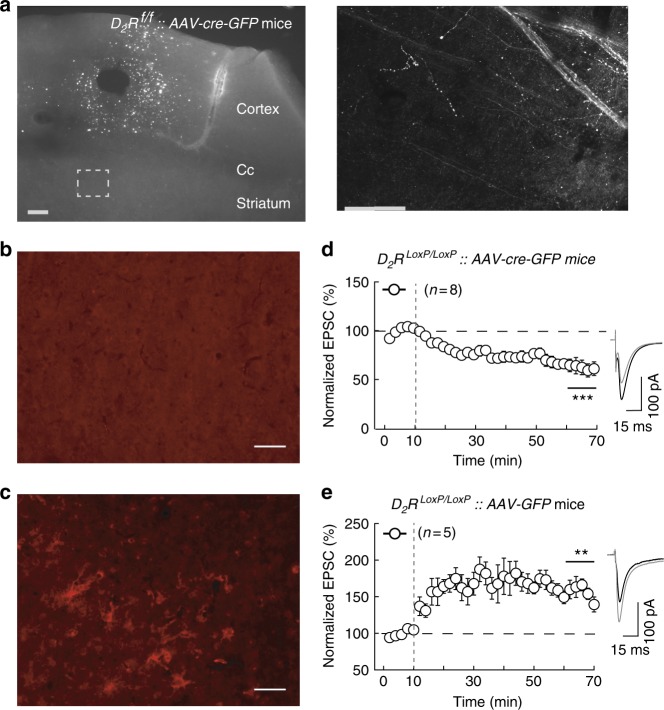
eCB-tLTP depends on D_2_R expressed by cortical pyramidal cells. **a** GFP fluorescence in D_2_R^LoxP/LoxP^ injected with AAV-cre-GFP in somatosensory cortex. Left panel: site of injection of the AAV-cre-GFP and the resulting fluorescence in pyramidal cells in somatosensory cortex (scale bar: 250 µm). Note that the fluorescence is restricted to neurons within cortex and does not cross corpus callosum (Cc). Right panel: GFP fluorescence in corticostriatal afferents within dorsal striatum (scale bar: 100 µm). **b**, **c** D_2_R immunostaining in the somatosensory cortex of D_2_R^LoxP/LoxP^ mice injected with AAV-cre-GFP (**b**) or with AAV-GFP (**c**); (scale bar: 100 µm). **d**, **e** Experiments of plasticity expression in cKO mice for D_2_R expressed at corticostriatal glutamatergic afferents. **d** 15 post-pre pairings failed to induce tLTP in D_2_R^LoxP/LoxP^ mice injected with AAV-cre-GFP in somatosensory cortex (*n* = 8, 0/8 and 7/8 cells showed tLTP and tLTD, respectively). **e** As a control, tLTP was induced with 15 post-pre pairings in D_2_R^LoxP/LoxP^ mice injected with AAV-GFP (*n* = 5, 5/5 cells showed tLTP). Representative traces are the average of 15 EPSCs during baseline (black traces) and 45 min after STDP protocol (grey traces). Vertical grey dashed line indicates the STDP protocol. Error bars represent sem. **p < 0.01; ***p < 0.001 by one sample *t*-test (**d**, **e**)

We next selectively inactivated D_2_R at corticostriatal glutamatergic afferents by cKO to test the implication of the D_2_R expressed by neocortical pyramidal cells in eCB-tLTP (Fig. 7). Bilateral KO of D_2_R in pyramidal cells was obtained by the stereotaxic injection of *AAV-cre-GFP* (*AAV1.CMV.HI.eGFP-Cre.WPRE.SV40*) in the layer 5 of the somatosensory cortex of *D*_*2*_*R*^*LoxP/LoxP*^ mice (Fig. 7a). In *D*_*2*_*R*^*LoxP/LoxP*^ mice injected with *AAV-cre-GFP*, we did not observe detectable positive immunostaining for D_2_R expression in pyramidal cells (Fig. 7b), whereas positive cells were observed in *D*_*2*_*R*^*LoxP/LoxP*^ mice injected with *AAV-GFP* (Fig. 7c); these later mice serving as control. Whole-cell recordings of MSNs from *D*_*2*_*R*^*LoxP/LoxP*^ mice injected bilaterally with *AAV-cre-GFP*, showed that 15 post-pre pairings failed to induce tLTP, but tLTD was observed instead (60 ± 7%, *p* = 0.0008, *n* = 8; Fig. 7d). As a control, we similarly injected *AAV-GFP* in *D*_*2*_*R*^*LoxP/LoxP*^ mice and observed tLTP induced by 15 post-pre pairings (150 ± 8%, *p* = 0.0025, *n* = 5) (Fig. 7e). These results demonstrate that eCB-tLTP depends on presynaptic D_2_R-activation in cortical terminals.

### Dopamine and eCB-tLTP interaction: a mathematical model

**Fig. 8:**
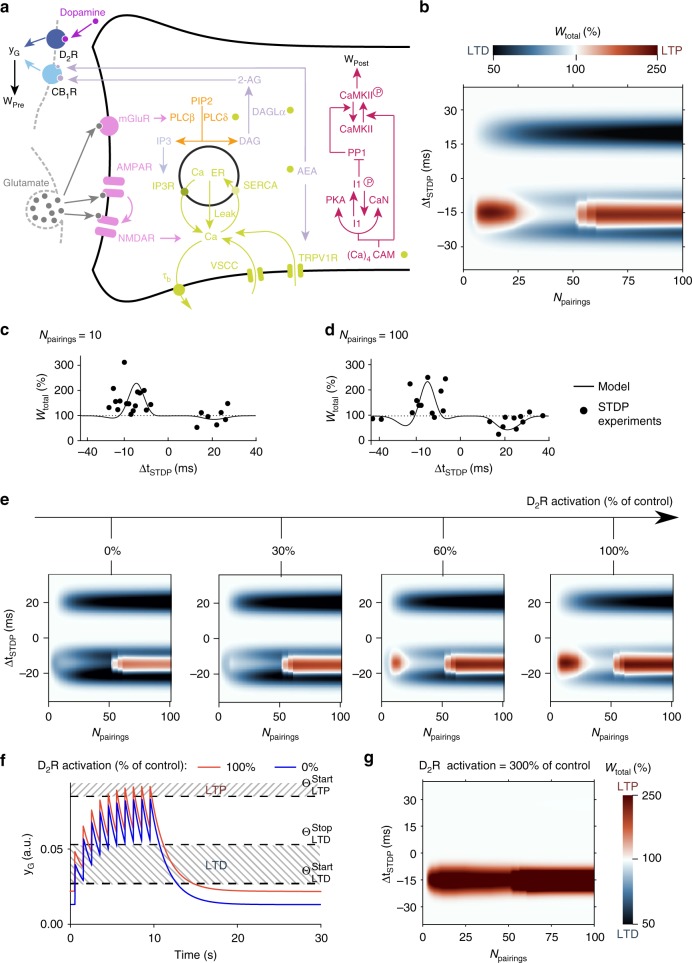
Threshold-based model interaction between dopamine and eCB-STDP. **a** Scheme of the modeled signaling network. The NMDAR-based pathway sets the postsynaptic weight *W*_post_ as the phosphorylation state of CaMKIIα. In the second pathway, coincident activation of phospholipase-Cβ by postsynaptic mGluR and calcium entry via VSCC and TRPV1 induces eCB production (2-AG and AEA). eCBs activates CB_1_R which modulates *W*_pre_, and *W*_total_ = *W*_post_ × *W*_pre._ In the model, D_2_R in the presynaptic cortical neurons co-localize with CB_1_R, so the effects of D_2_R and CB_1_R activations cumulate to determine whether the threshold for eCB-tLTP is reached. As a result, D_2_R activation by dopamine also contributes to *W*_pre_ changes_._ Green disks indicate calcium-dependent steps. For a thorough description, see ref. ^13^. Abbreviations: PIP2 phosphatidylinositol 4,5-biphosphate; DAG diacylglycerol; IP3 inositol-1,4,5-triphosphate; PLCβ/δ phospholipase-Cβ/δ; DAGLα diacylglycerol lipase-α; 2-AG 2-arachidonoylglycerol; AEA anandamide; IP3R IP3-receptor channel; SERCA sarcoplasmic/endoplasmic reticulum calcium ATPase; Ca_ER_ calcium in the endoplasmic reticulum; (Ca)_4_CaM fully bound calmodulin; CaN calcineurin aka PP2B; I1p/I1 phosphorylated/unphosphorylated protein phosphatase-1 inhibitor-1 (DARPP-32 in MSNs); PP1 protein phosphatase-1; CaMKII Ca^2+^/calmodulin-dependent protein kinase-II. **b** Model prediction for *W*_total_ changes (LTP and LTD) with varying *N*_pairings_ and Δ*t*_STDP_ (at 1 Hz) shows eCB-tLTP (3 < *N*_pairings_ < 40, −10 < Δ*t*_STDP_ < −25 ms) and the eCB-tLTD domains (*N*_pairings_ > 70, +10 < Δ*t*_STDP_ < +25 ms). **c**, **d** Changes of *W*_total_ with Δ*t*_STDP_, for *N*_pairings_ = 10 (**c**) or 100 (**d**) pairings at 1 Hz. Black lines are simulation results whereas the black circles show experimental measurements. **e** *W*_total_ change map of **b** when presynaptic D_2_R activation is reduced from 100 to 0% of the control (shown in **b**). **f** Temporal evolution of *y*_G_, that combines the effects of D_2_R and CB_1_R activation during STDP protocol (10 pairings at 1 Hz, Δ*t*_STDP_ = −15 ms). LTD is triggered when *y*_G_ is between ${\mathrm{\theta }}_{{\mathrm{LTD}}}^{{\mathrm{start}}}$ and ${\mathrm{\theta }}_{{\mathrm{LTD}}}^{{\mathrm{stop}}}$ whereas LTP is triggered when *y*_G_ > ${\mathrm{\theta }}_{{\mathrm{LTP}}}^{{\mathrm{start}}}$. D_2_R activation was 100% (red full line) or 0% (blue full line). **g** Model prediction for *W*_total_ upon hyperdopaminergy, with a threefold increase of tonic dopamine (DA, see Supplementary Experimental Procedures) compared to the control case shown in **b**. All other parameters were as in **b**

To provide hypotheses for the effects of the D_2_R inhibition/deletion/cKO (Figs. 4–7), we developed a biologically plausible mathematical model^13^ of corticostriatal synaptic plasticity. Our model emulates the temporal dynamics of the signaling pathways involved in corticostriatal STDP^12,13,28,29,31,32^, combining a pathway leading from NMDAR to calmodulin and CaMKII with a second one that links postsynaptically mGluR5 and cytosolic calcium to eCB production and results in the activation of presynaptic CB_1_R (Fig. 8a). Our model expresses chemical kinetics for the reactions in these two pathways (Fig. 8a). Repeated pairing stimulations of the pre- and postsynaptic neurons trigger transient changes of the species implicated in the biochemical reaction network illustrated (Fig. 8a) according to standard mass-action law kinetics. Hence, the temporal evolution of each species emerges from the coupling between the reaction network and the stimulations. We used the amount of phosphorylated postsynaptic CaMKII as a proxy for the postsynaptic contribution to the synaptic weight, while presynaptic G_i/o_-GPCR activation by CB_1_R and D_2_R was taken as a proxy for the presynaptic contribution (see Methods). The total synaptic weight (*W*_total_) was computed as the product of pre- and postsynaptic contributions^13^. Our model accounts for the outcome of plasticity when the spike timing (Δ*t*_STDP_), the frequency (*F*_pairings_), and the number (*N*_pairings_) of pairings are varied. The *W*_total_ changes for *F*_pairings_ = 1 Hz and a range of −35 < Δ*t*_STDP_ < +35 ms and for 1 < *N*_pairings_ < 100 values are illustrated by the color-map of Fig. 8b. In agreement with experimental data^12,13^, the outcome of plasticity is splitted along three plasticity domains: a first tLTP (i.e., eCB-tLTP) domain for −3 < Δ*t*_STDP_ < −25 ms and 3 < *N*_pairings_ < 30, a second tLTP (i.e., NMDAR-tLTP) domain for −10 < Δ*t*_STDP_ < −25 ms and *N*_pairings_ > 50, and a tLTD (i.e., eCB-tLTD) domain for 10 < Δ*t*_STDP_ < 25 ms and *N*_pairings_ > 20. The quality of the match between model prediction and electrophysiology measurements is illustrated in Fig. 8c, d (see ref. ^13^, for a more thorough account).

We next examined the dependence of plasticity on the activation of dopamine receptors, since our model integrates the dependence of plasticity on presynaptic D_2_R (see Methods). We compared the changes of eCB-tLTP domain under presynaptic D_2_R inhibition in the model against the data. Figure 8e illustrates the changes experienced by the two eCB-controlled domains when the level of presynaptic D_2_R activation decreases progressively from 100 to 0% of its control value (*γ*_D2R_ = 0.84, 0.50, 0.25, and 0.00; see Methods). The eCB-tLTD domain (for Δ*t*_STDP_ around +20 ms) is not affected by the decrease of presynaptic D_2_R signaling. In contrast, eCB-tLTP (centered on *N*_pairings_ = 15 and Δ*t*_STDP_ = −15 ms) is altered by a reduction of presynaptic D_2_R signaling: eCB-tLTP magnitude first decreases and is then replaced by eCB-tLTD. Therefore, our model predicts that blockers of presynaptic D_2_R do not affect eCB-tLTD and convert eCB-tLTP into tLTD; the latter prediction matches with the experimental data (Figs. 3f and 7d).

We next analyzed the dynamics of the model to explain this progressive change of eCB-tLTP to no plasticity then to eCB-tLTD as D_2_R inactivation increases. The variable *y*_*G*_ summarizes the effects of CB_1_R and D_2_R activation on the presynaptic weight (*W*_pre_). We assumed that the effects of D_2_R and CB_1_R are cumulative, up to the tLTP threshold. We expressed this assumption mathematically by the linear combination of three terms: *y*_*G*_ = *k*_CB1R_⋅*x*_CB1R_ + *γ*_D2R_⋅DA + *γ*_other_. The first term, presynaptic CB_1_R activation (*x*_CB1R_) is controlled by postsynaptically-produced eCBs (see Methods), the dynamics of which comprises calcium-dependent biochemical reactions. Since postsynaptic calcium dynamics in our model emerges from the interaction between the paired stimulations and the biochemical network, *y*_*G*_ dynamics indirectly depends on spike timing via the calcium-dependence of *x*_CB1R_. The second term depends on D_2_R activation (*γ*_D2R_⋅DA) and, the third one, *γ*_other_ accounts for background activation of the G_i/o_-GPCR pathway not related to D_2_R and CB_1_R.

Here, *W*_pre_ is set by *y*_*G*_ relatively to three plasticity thresholds ($\theta _{{\mathrm{LTD}}}^{{\mathrm{start}}}$, $\theta _{{\mathrm{LTD}}}^{{\mathrm{stop}}}$, and $\theta _{{\mathrm{LTP}}}^{{\mathrm{start}}}$): *W*_pre_ drops (LTD) when *y*_*G*_ is in between $\theta _{{\mathrm{LTD}}}^{{\mathrm{start}}}$ and $\theta _{{\mathrm{LTD}}}^{{\mathrm{stop}}}$, whereas *W*_pre_ rises (LTP) if *y*_*G*_ becomes larger than $\theta _{{\mathrm{LTP}}}^{{\mathrm{start}}}$ (Fig. 8f, dashed line). In control conditions (Fig. 8f, red line), the combination of the effect of CB_1_R activation by eCBs (produced upon the STDP protocol) and that of D_2_R by (tonic) dopamine, build up to yield *y*_*G*_ levels (red) that overcome $\theta _{{\mathrm{LTP}}}^{{\mathrm{start}}}$ resulting in LTP. However, with D_2_R activation blocked (Fig. 8f, blue line), *y*_*G*_ cannot reach $\theta _{{\mathrm{LTP}}}^{{\mathrm{start}}}$, thus effectively preventing the expression of eCB-tLTP. Nevertheless, *y*_*G*_ still crosses the LTD range with LTD accumulating in proportion to the time spent between ${\mathrm{\theta }}_{{\mathrm{LTD}}}^{{\mathrm{start}}}$ and ${\mathrm{\theta }}_{{\mathrm{LTD}}}^{{\mathrm{stop}}}$. As a result, eCB-tLTD is expressed instead of eCB-tLTP. This is in agreement with our experimental data (Figs. 3f and 7d) in which the prolonged inhibition or deletion of D_2_R switched eCB-tLTP into tLTD, whereas brief (16 s) opto-inhibition prevented eCB-tLTP without conversion into tLTD (Fig. 3c). Therefore, according to the model, the switch from eCB-tLTP to eCB-tLTD observed when presynaptic D_2_R are blocked is due to the reduction of the synergestic effect of D_2_R on the presynaptic terminal that needs to be present in addition to the activation of the presynaptic G_i/o_-GPCR pathway by CB_1_R to reach $\theta _{{\mathrm{LTP}}}^{{\mathrm{start}}}$. Thus, the presynaptic cortical D_2_R not only allow the expression of eCB-tLTP, but even control the polarity (LTP vs LTD) of the plasticity induced by a low number of pairings.

The above results (experimental and theoretical) consider 1 Hz stimulations. In the neocortex, increasing *F*_pairings_ with large numbers of pairings promotes tLTP at the expense of tLTD^49^. We next used the model to address the effects of D_2_R block on STDP when *F*_pairings_ varies (Supplementary Fig. 6). Note that our model was calibrated based on experimental data harvested at 1 Hz and does not account for the complex frequency-dependence of glutamate signaling (glutamate release and uptake, AMPAR desensitization). For this reason, our previous investigations^13^ have shown that sensitivity to frequency changes is larger in the model than in the experiments, so that the effects of a small change of *F*_pairings_ in the model (1.00–1.05 Hz) are similar to the effects of larger changes (1–3 Hz) in the experiments (see Discussion). In control conditions (100% D_2_R activation) and small numbers of pairings (*N*_pairings_ < 25 and Δ*t*_STDP_ < 0), eCB-tLTP is not expressed when *F*_pairings_ < 0.9 Hz. However, eCB-tLTP is not much altered above the threshold (Supplementary Fig. 6b, 6d and 6f). Note that eCB-tLTD observed at negative Δ*t*_STDP_ (blue areas in Supplementary Fig. 6b) is in all case negligible in control conditions for *N*_pairings_ < 25. When presynaptic D_2_R are blocked (Supplementary Fig. 6a) the model shows a similar picture, but the frequency threshold for eCB-tLTP is larger than 1 Hz. This explains the disappearance of eCB-tLTP at 1 Hz when D_2_R are blocked (Supplementary Fig. 6a). Moreover the faint eCB-tLTD present in control conditions at Δ*t*_STDP_ < 0 is amplified in the absence of D_2_R activation (Supplementary Fig. 6a, blue areas for *N*_pairings_ < 25, Δ*t*_STDP_ < 0). This explains the emergence of eCB-tLTD at 1 Hz when D_2_R are blocked.

The impact of *F*_pairings_ on STDP is quite different with larger pairings (*N*_pairings_ > 50). In control conditions (100% D_2_R activation), the expression domain of NMDAR-tLTP (*N*_pairing_ > 50, Δ*t*_STDP_ < 0) enlarges drastically when *F*_pairings_ increases (Supplementary Fig. 6b and 6d). NMDAR-tLTP even invades the quadrant of positive Δ*t*_STDP_ (Supplementary Fig. 6f). This strengthening of NMDAR-tLTP with increased *F*_pairings_ matches published experimental reports^49,50^. In comparison, the expression of eCB-tLTD with such large pairings (*N*_pairings_ > 50, Δ*t*_STDP_ > 0) is not much altered by large frequencies. Since presynaptic D_2_R in the model only affects eCB plasticity, blocking D_2_R receptors has no effect on NMDAR-tLTP directly (Supplementary Fig. 6a, 6c and 6e, *N*_pairings_ > 50, Δ*t*_STDP_ < 0), although it strengthens eCB-tLTD, thus partially canceling NMDR-tLTP. To summarize, at *F*_pairings_>1 Hz our model predicts that presynaptic D_2_R signaling fine-tunes STDP polarity (LTP or LTD) via the control of eCB-plasticity.

Hyperdopaminergy in the striatum is of interest since observed in drug addiction^51^. We next used our mathematical model to explore the effects of hyperdopaminergy on STDP. Figure 8g shows model prediction when the level of tonic dopamine is increased threefold with respect to control. eCB-tLTP induction is hardly altered by hyperdopaminergy. The main modification is that larger *N*_pairings_ can induce eCB-tLTP_,_ so eCB-tLTP domain fuses with the NMDAR-tLTP domain. In contrast, hyperdopaminergy has a drastic effect on eCB-tLTD: the whole eCB-tLTD domain disappears so pre-post pairings fail to induce any plasticity regardless *N*_pairings_ or Δ*t*_STDP_ (Fig. 8g). With large D_2_R activation, *y*_*G*_ remains located between ${\mathrm{\theta }}_{{\mathrm{LTD}}}^{{\mathrm{stop}}}$ and ${\mathrm{\theta }}_{{\mathrm{LTP}}}^{{\mathrm{start}}}$, which is too large to induce LTD but too weak to trigger LTP. Therefore, the prediction of our model is that hyperdopaminergy (via activation of presynaptic cortical D_2_R), should prevent eCB-tLTD and considerably extend the domain of expression of eCB-tLTP.

## Discussion

Corticostriatal long-term plasticity provides a fundamental mechanism for the function of the basal ganglia in procedural learning^14–19,21,52^. We uncovered the existence of striatal eCB-tLTP that relies on of CB_1_R and TRPV1 activation and is induced by a low number of pairings^12,13^. eCB-tLTP requires high levels of CB_1_R activation that can be reached with 10–15 post-pre pairings. Indeed, for such *N*_pairings_, eCB synthesis and release would contribute maximally to presynaptic CB_1_R activation (maximal cytosolic calcium influx from NMDAR, VSCC, TRPV1, and maximal calcium efflux from internal stores, combined with a minimal CB_1_R desensitization)^13^. According to our mathematical model, beyond 30 post-pre pairings, calcium efflux from the internal calcium stores decreases (because of a sub-optimal calcium refilling of the internal stores) while CB_1_R desensitization increases concomitantly. CB_1_R activation then crosses $\theta _{{\mathrm{LTP}}}^{{\mathrm{start}}}$ (Fig. 8f), so that eCB-tLTP vanishes, and NMDAR-tLTP can be expressed for post-pre *N*_pairings_ > 50 (Fig. 8b; see ref. ^13^). Interestingly, GABAergic microcircuits are not involved in eCB-tLTP induction or magnitude at corticostriatal synapses, but control eCB-tLTP polarity^12^. eCBs have been mainly reported to depress synaptic weight through the activation of CB_1_R or TRPV1^1,2^. This view is now challenged by studies reporting opposite effects (potentiation) in various brain structures using various signaling pathways^3–13^. We describe here and in previous studies^11–13,50^ a tLTP in mammals, wherein eCB signaling directly underlies both the induction and the long-term maintenance of synaptic weight increase.

Our mathematical model is sensitive to *F*_pairings_ (Supplementary Fig. 6). The results predicted by the model for small frequency changes (from 1.0 to 1.2 Hz) match well the results we obtained experimentally with larger frequency changes (from 1 to 3 Hz). This distortion may be due to the numerous frequency-dependent mechanisms (glutamate release or uptake, AMPAR desensitization) that buffer the effects of *F*_pairings_ increase in the experiments. For instance, the amount of glutamate released upon presynaptic stimulation does not depend on the frequency of presynaptic stimulation in the model. Therefore, the impact of increased frequency on the amplitude of the calcium trace is larger than in the experiments where presynaptic short-term plasticity decreases the amount of released glutamate when presynaptic frequency increases^53^, thus compensating for the effects of frequency increase on the calcium traces. Those frequency-dependent mechanisms could have been accounted for but would have been at the expense of a further complexification of the model.

Dopamine is a key actor of action selection and associative learning^22^ and for the modulation of striatal projection^24,25,33^. Electrical, chemical, or transcranial stimulation in different cortical areas enhances dopamine release in the corresponding projecting striatal areas including the dorsal striatum^38–40^. Our results show that dopamine is a key element for eCB-tLTP expression through the activation of D_2_R. Contrarily to eCB-LTD induced with high- or low-frequency stimulation protocol^24,25^ (i.e., a rate-coding paradigm), eCB-tLTP (induced here with a low number of STDP pairings, i.e., a time-coding paradigm) does not require postsynaptic D_2_R expressed by striatopallidal neurons. Indeed, the selective ablation of those D_2_R-MSNs or the selective D_2_R cKO in D_2_R-MSNs failed to prevent eCB-tLTP. Moreover, we observed eCB-tLTP in D_1_R-expressing MSNs as well as D_1_R-non-expressing cells. This demonstrates that D_2_R involved in eCB-tLTP have a presynaptic location. The presynaptic D_2_R are expressed at three different locations: the nigrostriatal dopaminergic afferents^45^, the cholinergic interneurons^44^, and the corticostriatal glutamatergic afferents^46,47^. Presynaptic D_2_R in nigrostriatal dopaminergic afferents and cholinergic interneurons are not required for eCB-tLTP as demonstrated by ablation or selective D_2_R cKO experiments. Although eCB-tLTP is muscarinic M_1_R-mediated^13^, eCB-tLTP was observed with ablated cholinergic interneurons suggesting that M_1_R should have a constitutive activity^54^. Moreover, the involvement of D_2_R expressed by cholinergic interneurons is unlikely since the activation of those D_2_R would decrease acetylcholine release, promoting LTD, and not LTP^55^. eCB-tLTP was absent in mice in cKO of D_2_R in the somatosensory cortex. Those results unambiguously demonstrate that D_2_R implicated in the control of eCB-tLTP are located on the presynaptic pyramidal neurons, where the CB_1_R are also expressed. Interestingly, eCB-LTD (rate-coded) has been reported to be tightly controlled by D_2_R expressed by striatal cholinergic interneurons^55,56^ or by D_2_R-MSNs^57,58^ (for review see ref. ^24^), i.e., distinct pools of D_2_R are engaged in eCB-LTD and eCB-LTP. In the nucleus accumbens, activation of CB_1_R on cortical terminals limits dopamine release and governs reward-driven behavior^59^. Dopamine–eCB interactions remain to be examined in dorsal striatum in the temporal-credit assignment problem^60–62^.

Colocalization of D_2_R and CB_1_R on the same presynaptic terminals would act synergistically on adenylate cyclase activity and promote eCB-tLTP or eCB-tLTD induced by small numbers of pairings, depending on the activation of D_2_R and CB_1_R. Our results suggest a joint CB_1_R- and D_2_R-control of STDP whereby the same signal, adenylate cyclase or PKA activity, can trigger tLTP or tLTD depending on the dynamics of the signaling pathway^13^. A similar hypothesis has been proposed for NMDAR-dependent STDP where the outcome of plasticity depends on the dynamics of intracellular calcium^63,64^. However our results unravel further complexity of the system, since in our hands the eCB-tLTD induced with 100 pairings shows additional dependence on D_1_R (both D_2_R- and D_1_R-mediated). This result matches some of the earlier results on corticostriatal STDP^28^; but see the STDP with 100 pairings at 0.1 Hz in which tLTD was D_1_R- but not D_2_R-dependent^29^. We observed that in both cases, pharmacological blocking of D_2_R gave rise to a switch of plasticity: with 100 pre-post pairings, tLTP was obtained instead of eCB-tLTD, whereas with 10 post-pre pairings D_2_R inhibition resulted in tLTD instead of eCB-tLTP; note that the inhibition of D_1_R has no effect on eCB-tLTP and prevents eCB-tLTD. Interestingly, only prolonged inhibition of D_2_R (pharmacological and genetic deletion experiments) switched eCB-tLTP into tLTD whereas a brief (16 s) opto-inhibition of dopamine release during STDP pairings prevented eCB-tLTP without inducing tLTD. We hypothesize that opto-inhibition likely prevents phasic dopamine release during STDP pairings without affecting much background tonic dopamine levels whereas prolonged inhibition of D_2_R would affect preferentially the tonic component. D_2_R have been widely described to efficiently sense the tonic dopamine because of their high affinity for dopamine^65^, but D_2_R can also encode phasic dopamine signals^66^. This suggests that depending on the level of the tonic dopamine, D_2_R would favor either eCB-tLTP or eCB-tLTD (for a low number of pairings). Thus, presynaptic cortical D_2_R operate as gatekeepers for the expression and polarity of corticostriatal eCB-STDP (eCB-tLTP and eCB-tLTD) depending on the activity pattern. This could be linked to the role of dopamine in the temporal credit-assignment by operating a retroactive control of STDP polarity, as demonstrated in striatum^60–62^ or in hippocampus^67^.

Although, eCB-tLTP can be induced with 15 post-pre pairings in D_1_R- and D_2_R-MSNs, it does not imply that in physiological conditions eCB-tLTP would be equally triggered in these MSN subpopulations. Indeed, D_2_R-MSNs are more excitable than D_1_R-MSNs^68^ and thus would not integrate similarly incoming cortical inputs, especially in the frame of a plasticity induced by a very low number of pairings, such as eCB-tLTP. This differential membrane excitability may be exacerbated by a higher excitability of D_2_R-MSN dendrites, allowing back-propagating action potentials to invade more efficiently distal dendrites in D_2_R-MSNs^69^. This is of importance for STDP in which a back-propagating action potential is triggered for every pairings. Therefore, one can expect that eCB-tLTP would be more prone to occur in D_2_R- than in D_1_R-MSN. It thus remains to investigate whether D_2_R-MSN would have a proeminent role in the fast learning via expression of eCB-tLTP. The use of noisy STDP pairings, to approach in-vivo-like conditions, revealed that plasticity robustness depends on the signaling pathways with eCB-tLTP and eCB-tLTD being much more robust than NMDAR-tLTP^50^. In vivo conditions for eCB-tLTP emergence, using naturalistic firing patterns recorded across learning tasks still need to be determined.

Alterations of the eCB system seem to contribute to action-learning defects in Parkinson’s disease^24,34^. So far, this alteration was thought to rely on disruption of the well-characterized eCB-LTD, which is D_2_R-dependent^24,25^. Here, we report that eCB-tLTP is also D_2_R-dependent, is disrupted in a rodent model of Parkinson’s disease, and can be rescued with L-DOPA treatment. D_2_R are central in various psychiatric diseases^70^. According to our findings, the effect of dopamine on corticostriatal STDP would vary with the cellular location (MSN, cholinergic, dopaminergic, or neocortical neurons) of D_2_R and on the thalamus/cortical activity regimes. It remains to analyze the effects of the disruption of eCB-tLTP in vivo to address the roles of eCB-tLTP in physiological and pathophysiological states. From our mathematical model, it is expected that the eCB-tLTP expression domain extends considerably under hyperdopaminergia. One could thus speculate that psychostimulant drugs such as cocaine or amphetamines (which trigger a hyperdopaminergia) would promote eCB-tLTP and possibly facilitate the early phase of learning. On the contrary, upon hypodopaminergia, it is expected that LTD will take over instead of eCB-tLTP and thus affect the fast learning. These predictions remain to be tested in the frame of learning and memorizing salient events from a low number of spikes^20^. Because it is induced by small numbers of pairings, eCB-tLTP may represent a central molecular substrate for the rapid learning of new arbitrary associative memories and behavioral rules^20^ characterizing the flexible behavior of mammals or during initial stages of slower habit learnings^14,52^, which may possibly be disrupted in Parkinson’s disease^24,34^.

## Methods

### Animals

Sprague–Dawley rats (Charles River, L’Arbresle, France) and C57BL/6 mice (*DAT-Cre*, *Archeorhodopsin3-GFP*, *Adora2a-Cre*, *inducible-DTR*, *Drd1a-GFP*, *ChAT-cre* and *D*_*2*_*R*^*LoxP/LoxP*^, *A*_*2A*_*-Cre*^+/−^*::Drd2*^*LoxP/LoxP*^, *ChAT-Cre*^+/−^*::Drd2*^*LoxP/LoxP*^, *DAT-Cre*^+/−^*::Drd2*^*LoxP/LoxP*^ mice) were used for electrophysiology, in situ hybridization, and immunohistochemistry. *Adora2a-Cre*^+/−^ transgenic mice expressing the Cre recombinase under the control of the striatopallidal specific adenosine A2A receptor (*Adora2a*) promoter^48^. C57BL/6 *Adora2a-Cre*^+/−^ mice were crossed with C57BL/6 *iDTR*^*+/+*^ mice^71^, and Drd1a-GFP^+/+^ resulting in 50% triple-heterozygous *Adora2a-Cre*^+/−^*::iDTR*^+/−^*::D*_*1*_*R-GFP*^+/−^ and 50% *Adora2a-Cre*^*−/−*^*::iDTR*^+/−^*::D*_*1*_*R-GFP*^+/−^ which were used as controls. C57BL/6 *ChAT-Cre*^+/−^ (JAX SN6410) mice were crossed with C57BL/6 *iDTR*^*+/+*^ mice^71^. Drd2 floxed mice carrying two targeted loxP sites flanking Drd2 exon 2^72^. The Cre-mediated expression of Arch3 was obtained using mice expressing Cre-recombinase under the control of the Slc6a3 promoter (*DAT-Cre* mice, JAX SN006660) and *Rosa26 LSL Arch3-GFP* (Jackson Laboratory). Mice were housed by groups of 3–5 mice, in a 12 h light/dark cycle, with food and water available ad libitum. All experiments were performed in accordance with local animal welfare committee (CIRB and ULB Ethical Committees) and EU guidelines (Directive 2010/63/EU). Every precaution was taken to minimize stress and the number of animals used in each series of experiments.

### Patch-clamp recordings and analysis

Corticostriatal connections (between somatosensory cortex layer 5 and dorsal striatum) are preserved in a horizontal plane^30^. Horizontal brain slices with thickness of 330 or 300 μm were prepared, respectively, from rats (P_(20–25)_ for most experiments, P_50_ for 6-OHDA lesion, and P_(60–65)_ for Parkinson’s disease animal model) or mice (P_(25–45)_) using a vibrating blade microtome (VT1200S, Leica Microsystems, Nussloch, Germany). In a subset of experiments, we performed coronal brain slices to record in cell-attached dopaminergic neurons from the SNc. Brains were sliced in a 95% CO_2_/5% O_2_-bubbled, ice-cold cutting solution containing (in mM) 125 NaCl, 2.5 KCl, 25 glucose, 25 NaHCO_3_, 1.25 NaH_2_PO_4_, 2 CaCl_2_, 1 MgCl_2_, 1 pyruvic acid, and transferred into the same solution at 34 °C for 1 h and next moved to room temperature.

Patch-clamp recordings were performed in the dorsolateral striatum^12,32,35^. Borosilicate glass pipettes of 4–6 MΩ resistance contained for whole-cell recordings (in mM): 105 K-gluconate, 30 KCl, 10 HEPES, 10 phosphocreatine, 4 ATP-Mg, 0.3 GTP-Na, 0.3 EGTA (adjusted to pH 7.35 with KOH). The composition of the extracellular solution was (mM): 125 NaCl, 2.5 KCl, 25 glucose, 25 NaHCO_3_, 1.25 NaH_2_PO_4_, 2 CaCl_2_, 1 MgCl_2_, 10 μM pyruvic acid bubbled with 95% O_2_ and 5% CO_2_. Signals were amplified using EPC10-2 amplifiers (HEKA Elektronik, Lambrecht, Germany). All recordings were performed at 34 °C using a temperature control system (Bath-controller V, Luigs & Neumann, Ratingen, Germany) and slices were continuously superfused at 2–3 ml/min with the extracellular solution. Slices were visualized on an Olympus BX51WI microscope (Olympus, Rungis, France) using a 4×/0.13 objective for the placement of the stimulating electrode and a 40×/0.80 water-immersion objective for localizing cells for whole-cell recordings. Current- and voltage-clamp recordings were filtered at 5 kHz and sampled at 10 kHz using the Patchmaster v2x32 program (HEKA Elektronik).

Off-line analysis was performed using Fitmaster (Heka Elektronik) and Igor-Pro 6.0.3 (WaveMetrics, Lake Oswego, OR, USA). Statistical analysis was performed using Prism 5.0 software (San Diego, CA, USA). In all cases “*n*” refers to the number of repetitions of an experiment (each experiment being performed on different brain slices) from single slice. Experimenters were blind to most of the mice genotype during electrophysiological recordings and analysis. All results were expressed as mean ± sem, and statistical significance was assessed using Mann–Whitney test or the one sample *t* test when appropriate at the significance level (*p*) indicated.

### Spike-timing-dependent plasticity induction protocols

Electrical stimulation was performed with a bipolar electrode (Phymep, Paris, France) placed in the layer 5 of the somatosensory cortex^12,30^. Electrical stimulation was monophasic at constant current (ISO-Flex stimulator, AMPI, Jerusalem, Israel). Currents were adjusted to evoke 50–200 pA EPSCs. Repetitive control stimuli were applied at 0.1 Hz. STDP protocols consisted in pairings of pre- and postsynaptic stimulations (at 1 Hz) separated by a temporal interval (Δ*t*_STDP_). Presynaptic stimulations corresponded to cortical stimulations and the postsynaptic stimulation of an action potential evoked by a depolarizing current step (30 ms duration) in MSNs. MSNs were maintained all along the STDP experiments at a constant holding membrane potential which corresponds to their initial resting membrane potential (−81.6 ± 0.4 mV, *n* = 61). Thus, across experiment EPSCs were measured at the same membrane potential (voltage-clamp mode); STDP pairings (current-clamp mode) were conducted at this same holding membrane potential. Neurons were recorded for 10 min during baseline and for at least 50 min after STDP protocol; long-term synaptic efficacy changes were measured from 50 min. 30 successive EPSCs (at 0.1 Hz) were individually measured and averaged. Variation of input and access resistances, measured every 10 s all along the experiment, beyond 20% led to the rejection of the experiment.

STDP protocol consisting in 5–10 post-pre pairings (with a single postsynaptic spike) were sufficient to induce potent tLTP in rat while in C57BL/6 mice 15 pairings (with 2–3 postsynaptic spikes) were necessary to trigger tLTP.

### Chemicals

N-(piperidin-1-yl)-5-(4-iodophenyl)-1-(2,4-dichlorophenyl)-4-methyl-1H-pyrazole-3-carboxamide (AM251, 3 μM, Tocris) and (S-)-5-Aminosulfonyl-N-[(1-ethyl-2-pyrrolidinyl)methyl]-2-methoxybenzamide (sulpiride, 10 μM, Tocris) and (2*E*)-*N*-(2,3-Dihydro-1,4-benzodioxin-6-yl)-3-[4-(1,1-dimethylethyl)phenyl]-2-propenamide (AMG9810, 1 μM) were dissolved in ethanol and then added in the external solution at a final concentration of ethanol of 0.01–0.1%. R(+)-7-Chloro-8-hydroxy-3-methyl-1-phenyl-2,3,4,5-tetrahydro-1H-3-benzazepine hydrochloride (SCH-23390, 4 μM, Sigma), (4aR-trans)-4,4a,5,6,7,8,8a,9-Octahydro-5-propyl-1H-pyrazolo[3,4-g]quinoline hydrochloride (quinpirole, 10 μM, Tocris) and +N-(2,6-Dimethylphenylcarbamoylmethyl)triethylammonium chloride (QX-314, 10 μM, Tocris) were dissolved in water and added in the external solution. None of the bath-applied drugs had a significant effect on basal EPSC amplitudes: AM251 (3 μM): 101 ± 5% (*p* = 0.8610, *n* = 7), co-application of SCH23390 (4 μM) and sulpiride (10 μM): 102 ± 3%, (*p* = 0.5056, *n* = 9) and quinpirole (10 μM): 98 ± 2%, (*p* = 0.3449, *n* = 8).

### Opto-inhibition of dopaminergic neurons

We bred C57BL/6 *DAT-Cre*^+/−^ and C57BL/6 *Arch3-GFP*^*+/+*^ mice (Jackson Laboratory) leading to mice that selectively expressed Archeo-rhodopsin in DAT-expressing neurons (*DAT-Cre*^+/−^*::Arch3-GFP*^+/−^ mice); *DAT-Cre*^*−/−*^*::Arch3-GFP*^+/−^ mice were used as a control. 585 nm yellow-green light was delivered via field illumination using a high-power LED source (pE excitation System, CoolLED, Andover, UK).

Double immunostaining for GFP (for Arch3 expression in dopaminergic neurons) and TH. Brain slices (300 µm) were fixed overnight in 2% paraformaldehyde at 4 °C. Non-specific binding was blocked by incubating the slices for 2 h at room temperature in 10% normal goat serum (Merk-Millipore, Molsheim, France) in 2% BSA and 1% Triton X-100 solution. Brain slices were incubated with a pair of primary antibodies, chicken anti-GFP (1:2000, AB13970, Abcam, Paris, France) and mouse anti-TH (1:500, MAB318, Merk-Millipore), in 0.5% Triton X-100, 1% BSA and 2.5% normal goat serum (overnight 4 °C), and then with the secondary antibodies, goat anti-chicken Alexa (1:1000, A11039, Life Technology-Invitrogen, Villebon-sur-Yvette, France) and goat anti-mouse IgG1 (1:200, 1070–03, Southern Biotech-Clinisciences, Montrouge, France) (overnight 4 °C).

### Selective ablation of D_2_R-expressing MSNs

We bred C57BL/6 *Adora2a-Cre*^+/−^ and inducible C57BL/6 *DTR*^*+/+*^ (iDTR^+/+^) mice leading to double heterozygous: mice that selectively expressed the DTR in D_2_R-MSN (*Adora2a-Cre*^+/−^*::iDTR*^+/−^, *A*_*2*_*A-DTR*^+/−^ mice) and *Adora2a-Cre*^*−/−*^*::iDTR*^+/−^ (*A*_*2*_*A-DTR*^*−/−*^ mice) used as the corresponding control for electrophysiology^48^. A2A receptor promoter was chosen because specifically expressed in striatum only by D_2_R-MSNs^48^. Inducible ablation allows a high spatial resolution (achieved with stereotaxic injections of toxin) and prevents developmental adaptations. We next bred *Adora2a-Cre*^+/−^*::iDTR*^+/−^ mice with *Drd1a-GFP* mice^48^ to ensure recording D_1_R-MSNs. *Adora2a-Cre*^+/−^*::iDTR*^+/−^*::Drd1a-GFP*^+/−^ mice (and their control *Adora2a-Cre*^*−/−*^*::iDTR*^+/−^*::Drd1a-GFP*^+/−^*)*. At the age of 4 weeks, 1 μl of DT (Sigma-Aldrich) by side (diluted in PBS 0.01 M to a concentration of 100 pg/μl) was slowly stereotaxically injected at 0.25 μl/min with a blunt needle in four sides of the dorsal striatum with the following coordinates (with bregma as references): anterior +1.2 mm, lateral ±1.5 mm, ventral +3.6 mm; or anterior +0.5 mm, lateral ±1.8 mm, ventral +3.6 mm. We reported an almost complete loss of striatal A_2_A receptor binding from 14 days after DT injections^48^. Therefore, electrophysiological recordings were performed from 15–20 days after DT injections.

*In situ hybridization for enkephalin mRNA*: Striatal 20 μm coronal sections were cut from fresh-frozen brain (*Adora2a-Cre*^+/−^*::iDTR*^+/−^*::Drd1a-GFP*^+/−^ mice) and mounted on glass slides. After the hybridization for enkephalin mRNA procedure^48^, sections were exposed to Kodak Biomax MR film for 1–2 week(s) depending on the marker studied and digitalized images were generated from the autoradiograms. For quantification of in situ hybridization and binding (ImageJ software), an averaged optical density (OD) in different areas of interest was measured and background level was subtracted to obtain corrected values. Two to three sections were used for each animal to calculate the mean OD.

### Selective ablation of ChAT interneurons

We bred C57BL/6 *ChAT-Cre*^+/−^ and inducible C57BL/6 *DTR*^+/+^ (*iDTR*^+/+^) mice leading to double heterozygous: mice that selectively expressed the DTR in cholinergic interneurons (*ChAT-Cre*^+/−^*::iDTR*^+/−^, *ChAT-DTR*^+/−^ mice) and *ChAT-Cre*^*−/−*^*::iDTR*^+/−^ (*ChAT-DTR*^*−/−*^ mice) used as the corresponding control for patch-clamp experiments. The ablation is performed in the same condition than D_2_R-MSNs. 1 μl of DT (Sigma) by side (diluted in PBS 0.01 M to a concentration of 100 pg/μl) was stereotaxically injected at 0.25 μl/min with a blunt needle in four sides of the dorsal striatum with the following coordinates: anterior +1.2 mm, lateral ±1.5 mm, ventral +3.6 mm; or anterior +0.5 mm, lateral ±1.8 mm, ventral +3.6 mm. An almost complete loss of ChAT immunostaining in the striatum from 14 days after DT injections was observed and recordings were performed from 15–20 days after DT injections.

*ChAT immunostaining*: *ChAT-Cre*^+/−^*::iDTR*^+/−^ mice were anesthetized and perfused transcardially with 0.01 M PBS, followed by 4% paraformaldehyde for tissue fixation. Brains were dissected, dehydrated in 20–30% sucrose solutions and frozen. Coronal free-floating sections of 30 μm thickness were cut through the striatum with a freezing microtome (Leica). Sections were treated with 0.3% H_2_O_2_ in PBS (pH 7.4) 0.1% Triton X-100 for 45 min and non-specific antibody binding was blocked with normal horse serum in PBS (pH 7.4) 0.1%Triton X-100 for 60 min. Sections were incubated 48 h at 4 °C with the rabbit primary antibody: anti-ChAT (1/1000) (Chemicon) and then with donkey anti-rabbit biotinylated antibody (1/200, Jackson ImmunoResearch) and visualized with the ABC diaminobenzidine reaction.

### D_2_R cKO in D_2_R-MSN, cholinergic, and dopaminergic neurons

cKO mice were generated by crossing homologous *Drd2*^*LoxP*/*LoxP*^ with heterologous specific Cre^+/−^ driver mice and the resulting double heterologous *Cre*^*−/+*^::*Drd2*^*Wt/*/*LoxP*^ are crossed with heterologous *Drd2*^*Wt/*/*LoxP*^. This crossing gives the cKO Cre^+/−^::*Drd2*^*LoxP/LoxP*^, the Cre driver control Cre^+/−^::*Drd2*^*Wt/Wt*^ and the floxed gene control Cre^*−/−*^::*Drd2*^*LoxP/LoxP*^. The Cre-mediated deletion of *Drd2* in D_2_R-MSNs, striatal cholinergic interneurons, or dopaminergic neurons was obtained using mice expressing Cre-recombinase under the control of the *Adora2a* promoter (*Adora2a-Cre* mice^47^), *ChAT* promoter (*ChAT-Cre;* JAX SN6410), and *Slc6a3* promoter (*DAT-Cre* mice), respectively.

### Selective D_2_R cKO in neocortical pyramidal cells of layer 5

We stereotaxically injected, in somatosensory cortex layer 5, *AAV-cre-GFP* (AAV1.CMV.HI.eGFP-Cre.WPRE.SV40, UPENN Core) in C57BL/6 *D*_*2*_*R*^*LoxP/LoxP*^ mice leading to selective D_2_R-KO mice in corticostriatal cells; C57BL/6 *D*_*2*_*R*^*LoxP/LoxP*^ mice injected with AAV-GFP (AAV1.CMV.PI.eGFP.WPRE.bGH, UPENN Core) were used as the corresponding control. AAV-GFP and AAV-cre-GFP were stereotaxically injected at 0.1 μl/min with a blunt needle in four (bilaterally) sides of the somatosensory cortex with the following coordinates: anterior + 1.2 and +0.1 mm, lateral ±3.5 and ±3.8 mm, ventral −3.2 mm. Electrophysiological recordings were performed from 15–30 days after viral injection.

### D_2_R immunostaining

*D*_*2*_*R*^*LoxP/LoxP*^ mice were anesthetized and perfused transcardially with 0.01 M PBS, followed by 4% paraformaldehyde for tissue fixation. Brains were dissected, dehydrated in 20–30% sucrose solutions and frozen. Coronal sections of 16 μm thickness were cut through the striatum with a freezing microtome (Leica). Non-specific antibody binding was blocked with normal horse serum in PBS (pH 7.4) 0.1%Triton X-100 for 60 min. Sections were incubated 24 h at 4 °C with the rabbit primary antibody: anti-D_2_R (1/1000) (Frontier Science) and then with donkey anti-rabbit Alexa594 (1/200, Jackson ImmunoResearch).

### SNc or MFB 6-OHDA-lesioned rats and L-DOPA treatment

*6-OHDA-lesioned animals*: Sprague–Dawley rats weighing 125–150 g (Charles River Laboratories, L’Arbresle, France) were anesthetized with sodium pentobarbital (30 mg/kg i.p.; Ceva Sante Animale, Libourne, France) supplemented by injections of ketamine (27.5 mg/kg, im; Imalgène, Merial, Lyon, France). Thirty minutes before the injection of 6-OHDA (or vehicle in sham-operated animals), all animals received a bolus of desipramine (25 mg/kg, i.p.; Sigma-Aldrich) to prevent neurotoxin-induced damage of noradrenergic neurons. Animals were fixed in a stereotaxic frame (Kopf Instruments, Tujunga, CA, USA). Body temperature was maintained at 36.5 °C with a homeothermic blanket (Harvard Apparatus, Kent, UK). A small craniotomy was made unilaterally (left side) over the SNc or MFB and the overlying dura mater was removed. For the Parkinson’s disease model and for the removal of the nigrostriatal dopaminergic cells, an unilateral 6-OHDA lesion of the SNc and MFB were performed, respectively. A single stereotaxic injection of 6-OHDA (or of vehicle in the sham-operated animals) was delivered into the SNc on the left side (stereotaxic coordinates anteriority from the interaural line (A): 3.7 mm, laterality from the midline (L): 2.1 mm, depth from the cortical surface (H): −7.55 mm). Concerning the MFB lesion, 6-OHDA was injected into the left MFB; stereotaxic coordinates anteriority from the bregma (A): 4.5 mm, laterality from the midline (L): 1.2 mm, depth from the cortical surface (H): −7.9 mm. 6-OHDA (hydrochloride salt; Sigma) was dissolved immediately prior use in ice-cold 0.9% w/v NaCl solution containing 0.01% w/v ascorbic acid to a final concentration of 2.5 mg/ml. 4.0 μl of this 6-OHDA solution (or vehicle) was injected at a rate of 16 μl/h via a steel cannula (0.25 mm outside diameter) attached to a 10 μl Hamilton microsyringe (Cole-Parmer, London, UK) controlled by an electrical pump (KDS100; KD Scientific, Holliston, MA). After surgery, animals received an intramuscular injection of gentamicin to prevent bacterial infection (3 mg/kg, im; Gentalline, Schering-Plough, Levallois-Perret, France).

*Chronic L-DOPA treatment*: Two weeks after 6-OHDA injection, rats were splitted into two groups which received i.p. injection twice-daily for 10 days of either L-DOPA (10 mg/kg) with benzerazide (7.5 mg/kg) or saline. The sham-operated animals were also subjected to L-DOPA or saline injections with the same schedule. Animals received the last injection of L-DOPA or saline 30–60 min before being sacrified for ex vivo experiments.

*TH immunostaining*: The severity of the 6-OHDA lesions was quantified by striatal TH immunostaining. Brain slices were incubated in 0.1% H_2_O_2_ and 10% methanol in PBS (15 min) and then in a 1/500 dilution of mouse anti-TH monoclonal antibody (MAB318; Merk-Millipore) overnight at 4 °C. Biotin-goat anti-mouse secondary antibody (Life Technology-Invitrogen, Villebon-sur-Yvette, France) was incubated at a dilution of 1/500 for 2.4 h at room temperature and visualized using with avidin–biotin complex (ABC Elite standard, PK-4000, Vector Laboratories, Burlingame, CA, USA) and DAB detection kit (Vector Laboratories).

### Mathematical model

We used the mathematical model of corticostriatal synaptic plasticity^13^ with two additional parameters related to presynaptic plasticity (see below). This model implements a single dendritic spine and a soma as a single isopotential electrical compartment. Electrical activation is coupled to a detailed description of the postsynaptic signaling pathways from calcium currents to the activation of CaMKIIα and production of eCBs. Ordinary differential equations based on classical mass-action law formalisms are then used to express the corresponding reaction kinetics. Plasticity outcome is determined by *W*_total_ that has pre-and postsynaptic components and was computed as *W*_total_ = *W*_pre_⋅*W*_post_.

We modeled the glutamate concentration in the synaptic cleft, *G*(*t*), as a train of exponentially-decaying impulses triggered by presynaptic stimuli at time $t_{{\mathrm{pre}}_i}$:

1 $$\normalsize \it \normalsize G(t) = G_{max}\mathop {\sum}\limits_i {exp} \left( { - \frac{{t - t_{pre_i}}}{{\tau _G}}} \right)\Theta \left( {t - t_{pre_i}} \right)$$

where *G*_max_ is the peak glutamate concentrations and *τ*_*G*_ is the glutamate clearance rate. On the postsynaptic side, action currents resulting from postsynaptic stimulations were modeled according to:

2 $${I_{{\mathrm{action}}}\left( t \right) = - DC_{{\mathrm{max}}}\mathop {\sum }\limits_{\mathrm{i}} R\left( {t,t_{{\mathrm{post}}_i},DC_{{\mathrm{dur}}}} \right) - AP_{{\mathrm{max}}}\mathop {\sum }\limits_{\mathrm{i}} {\mathrm{\Theta }}(t - \delta - t_{{\mathrm{post}}_i}){\mathrm{exp}}\left( {\frac{{ - t + \delta + t_{{\mathrm{post}}_i}}}{{\tau _{{\mathrm{bAP}}}}}} \right)}$$

where the rectangle function *R*(*t*, *a*, *l*) = Θ(*t* − *a*) − Θ(*t* − *a* − *l*), *DC*_max_ and *DC*_dur_ are the amplitude and duration of step-current injected in the postsynaptic soma, *AP*_max_ is the amplitude of the action current producing the back-propagating action potential (bAP); *τ*_bAP_ is the timescale for bAP decay and *δ* accounts for the time elapsed between the onset of the postsynaptic step current and the action potential it triggers (~3 ms in MSNs). We modeled the electrical response to these stimulations in a postsynaptic element considered as a single isopotential compartment with AMPAR, NMDAR, VSCC, and TRPV1 conductances:

3 $$\begin{array}{*{20}{l}} {C_{\mathrm{m}}\frac{{dV}}{{dt}}} \hfill & = \hfill & { - g_L(V - V_{\mathrm{L}}) - I_{{\mathrm{AMPAR}}}(V,G(t)) - I_{{\mathrm{NMDAR}}}(V,G(t))} \hfill \\ {} \hfill & {} \hfill & { - I_{{\mathrm{VSCC}}}(V) - I_{{\mathrm{TRPV}}1}(V,AEA) - I_{{\mathrm{action}}}(t)} \hfill \end{array}$$

where *V* is membrane potential; *g*_*L*_ and *V*_*L*_ are leak conductance and reversal potential, respectively, and AEA stands for anandamide. For a detailed exposition of the models used for those currents, see refs. ^12,13^.

The dynamics of free cytosolic calcium *C* was computed according to:

4 $$T_{\mathrm{C}}(C)\frac{{dC}}{{dt}} = J_{IP_3R} - J_{{\mathrm{SERCA}}} + J_{{\mathrm{leak}}} + J_{{\mathrm{NMDAR}}} + J_{{\mathrm{VSCC}}} + J_{{\mathrm{TRPV}}1} - \frac{{C - C_{\mathrm{b}}}}{{\tau _{C_{\mathrm{b}}}}}$$

where the fluxes *J*_IP3R_, *J*_SERCA_, *J*_leak_ from and to the ER in the Calcium-Induced Calcium Release (CICR) system were taken from the model of De Pittà et al.^73^ (see also ref. ^12^) and *J*_NMDAR_, *J*_VSCC_, and *J*_TRPV1_ are the calcium fluxes from the plasma membrane channels (Eq. (3)). *C*_*b*_ is the basal cytosolic calcium level resulting from equilibration with calcium diffusion out of the cell and ${\mathrm{\tau }}_{{\mathrm{C}}_{\mathrm{b}}}$ is the corresponding timescale. *T*_*C*_(*C*) is a time scaling factor accounting for the presence of endogenous calcium buffers. See Cui et al.^12^ for details.

*W*_post_ was based on the activation by calcium of calmodulin and CaMKII and the regulation of this system by PKA, calcineurin and protein phosphatase 1 (PP1). The model of Cui et al.^12^ for this subsection is based on the biophysical model proposed in Graupner and Brunel^74^, that expresses the calcium-dependent kinetics of the protein complexes and enzymes along this pathway, including the calcium-dependent kinetics of CaMKII*, the activated form of CaMKII, that was assumed to set postsynaptic plasticity according to:

5 $$W_{post} = 1 + 3.5\frac{{CaMKII^ \ast }}{{CaMKII_{max}^ \ast }}$$

where $CaMKII_{{\mathrm{max}}}^ \ast$ is the maximal concentration of activate (phosphorylated) CaMKII.

Our model also accounts for the biochemical pathways leading to the production of the endocannabinoids 2-arachidonoylglycerol (2-AG) and AEA, and their subsequent CB_1_R activation. We modeled CB_1_R activation by 2-AG and AEA using a simple three-state kinetic model: open (*x*_CB1R_), desensitized (*d*_CB1R_) and inactivated (*i*_CB1R_).

6 $$\frac{{dx_{CB1R}}}{{dt}} = \alpha _{CB1R}eCB\left( {1 - d_{CB1R} - x_{CB1R}} \right) - \left( {\beta _{CB1R} + \gamma _{CB1R}} \right)x_{CB1R}$$

7 $$\frac{{dd_{CB1R}}}{{dt}} = - \varepsilon _{CB1R}d_{CB1R} + \gamma _{CB1R}x_{CB1R}$$

where *eCB* = *2-AG* + 0.10 *AEA* accounts for the act that AEA is a partial agonist of CB_1_R.

In the original model^13^, CB_1_R activation controls presynaptic plasticity via the CB_1_R-triggered G_i/o_-GPCR pathway. Here, we add the assumption that the effects of D_2_R and CB_1_R on *W*_pre_ are cumulative. We account here for the resulting synergistic effects by combining both effects linearly in the variable *y*_*G*_ that controls *W*_pre_:

8 $$y_G = k_{{\mathrm{CB}}1{\mathrm{R}}} \cdot x_{{\mathrm{CB}}1{\mathrm{R}}} + \gamma _{{\mathrm{D}}2{\mathrm{R}}} \cdot {\mathrm{DA}} + \gamma _{{\mathrm{other}}}$$

where *x*_CB1R_ is the fraction of activated (non-desensitized) CB_1_R, *k*_CB1R_ quantifies the effect of CB_1_R activation, DA is the concentration of (tonic) dopamine, *γ*_D2R_ quantifies the effect of D_2_R activation and *γ*_other_ summarizes the effects of other G_i/o_-GPCR pathways (beyond CB_1_R and D_2_R). Note that *y*_G_ was actually referred to as *y*_CB1R_ in ref. ^13^ whereas the term *γ*_D2R_⋅DA + *γ*_other_ was lumped in a constant *D*_1_. Finally, we assume that *y*_*G*_ controls *W*_pre_, which we express in the model via two functions Ω(*y*_*G*_) and $\tau _{W_{{\mathrm{pre}}}}(y_G)$:

9 $$\frac{{dW_{pre}}}{{dt}} = \frac{{{\mathrm{\Omega }}(y_G) - W_{pre}}}{{\tau _{W_{pre}}(y_G)}},$$

10 $$\tau _{W_{pre}}(y_G) = \frac{{P_1}}{{P_2^{P_3} + \left( {y_G + P_5} \right)^{P_3}}} + P_4,$$

11 $${\mathrm{\Omega }}(y_G) = \left\{ {\begin{array}{*{20}{l}} {1 + A_{LTP},} \hfill & {{\mathrm{if}}\,\theta _{{\mathrm{LTD}}}^{{\mathrm{start}}} < y_G < \theta _{{\mathrm{LTD}}}^{{\mathrm{stop}}},} \hfill \\ {1 - A_{LTD},} \hfill & {{\mathrm{if}}\,y_G > \theta _{{\mathrm{LTP}}}^{{\mathrm{start}}},} \hfill \\ {1,} \hfill & {{\mathrm{otherwise}}.} \hfill \end{array}} \right.$$

where *A*_*LTP*_, *A*_*LTD*_, *P*_1−5_ are positive constants; $\theta _{{\mathrm{LTD}}}^{{\mathrm{start}}}$, $\theta _{{\mathrm{LTD}}}^{{\mathrm{stop}}}$, and $\theta _{{\mathrm{LTP}}}^{{\mathrm{start}}}$ are plasticity thresholds. Here, Ω sets the steady-state presynaptic weight, while $1/\tau _{W_{pre}}$ sets the rate at which this steady-state is approached. Ω decreases (LTD) when *y*_*G*_ is in between the two threshold values, $\theta _{{\mathrm{LTD}}}^{{\mathrm{start}}}$ and $\theta _{{\mathrm{LTD}}}^{{\mathrm{stop}}}$ whereas Ω rises (LTP) if *y*_*G*_ becomes larger than $\theta _{{\mathrm{LTP}}}^{{\mathrm{start}}}$ (dashed line in Fig. 8c). Therefore, *W*_pre_ depends on *y*_*G*_ and *y*_*G*_ indirectly depends on cytoplasmic calcium, thus on spike timing. As a result, *W*_pre_ depends on spike timing in the model.

In the present study, we used *k*_CB1R_ = 2400; *γ*_D2R_ = 0.84, DA = 0.01 µM, *γ*_other_ = 12.6⋅10^−3^; *A*_LTD_ = 1.82, *A*_LTP_ = 24, $\theta _{{\mathrm{LTD}}}^{{\mathrm{stop}}} = 0.053$ and $\theta _{{\mathrm{LTP}}}^{{\mathrm{start}}} = 0.085$. For all other parameters, we used the same values as in ref. ^13^.

## Electronic supplementary material

Supplementary Information

## Data Availability

All relevant data are available from the authors. The mathematical model can be downloaded from https://senselab.med.yale.edu/modeldb/, accession #187605.
