# Supplementary material for: Dopamine–endocannabinoid interactions mediate spike-timing-dependent potentiation in the striatum
Source: Nat Commun. 2018 Oct 8;9:4118. doi: 10.1038/s41467-018-06409-5 (PMC6175920; doi:10.1038/s41467-018-06409-5)
Supplement: Supplementary file 1 — Supplementary Information [file 41467_2018_6409_MOESM1_ESM.pdf]

Supplementary information

Dopamine-endocannabinoid interactions mediate spike-timing-dependent potentiation in the striatum

Hao XU, Sylvie PEREZ, Amandine CORNIL, Bérangère DETRAUX, Ilya PROKIN, Yihui CUI, Bertrand DEGOS, Hugues BERRY, Alban de KERCHOVE d'EXAERDE and Laurent VENANCE

Content

Supplementary Figures 1-6

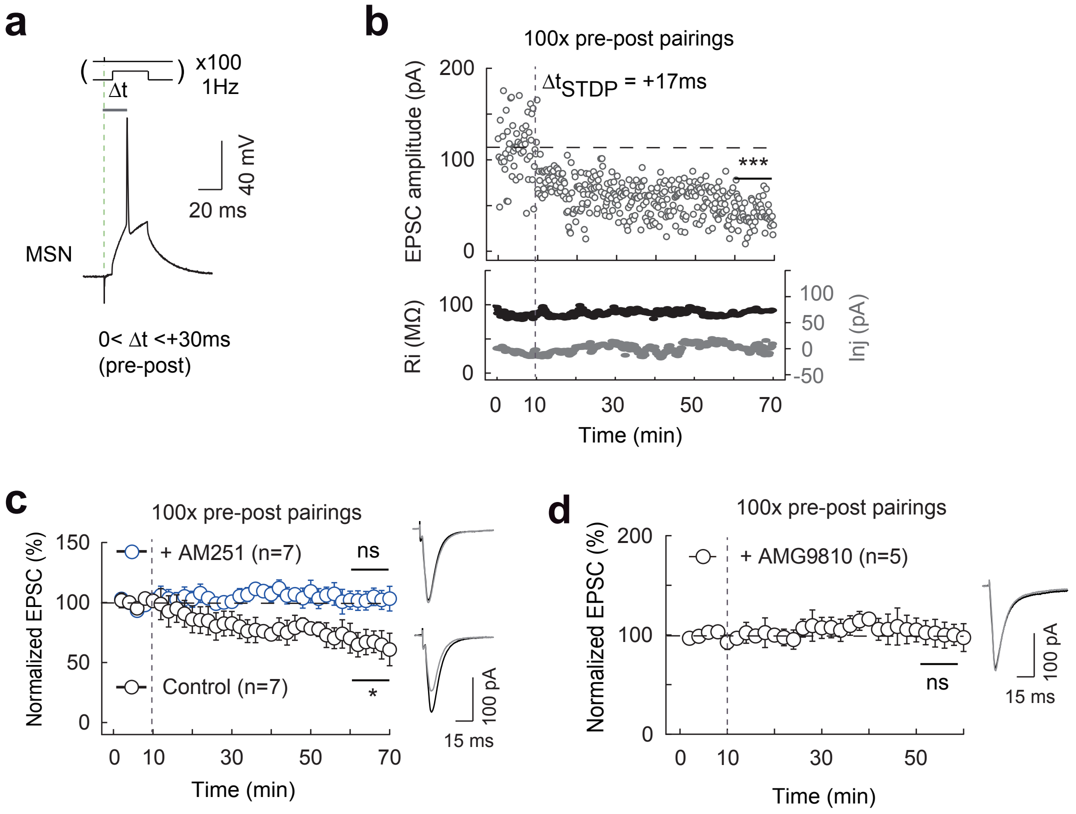

Supplementary Figure 1: Endocannabinoid-mediated-tLTD.

(**a**) STDP protocol: a spike evoked in one MSN was paired with a cortical stimulation repeated 100 times at 1Hz. Δt_{STDP} indicates the time shift between pre- and postsynaptic stimulations. $0<\Delta t_{STDP}<+30$ms refers to pre-post pairings. (**b-d**) tLTD induced with 100 pre-post pairings ($0<\Delta t_{STDP}<+30$ms) is CB_1R-activation dependent. (**b**) Example of tLTD induced by 100 pre-post pairings (Δt_{STDP}=+17ms). Top, EPSC strength before and after pairings (before pairings: 114±4pA; 55-60 min after pairings: 42±2pA; $p<0.0001$). Bottom, time courses of Ri (before, 83±0.6MΩ; after, 92±0.2MΩ; change of 11%) and injected current (Iinj) (before, -7±1pA; after, -0.5±2pA). (**c** and **d**) Summary of STDP experiments showing that tLTD induced with 100 pre-post pairings (n=7, 6/7 cells showed tLTD) is CB_1R- and TRPV1-mediated since prevented by AM251 (3µM, n=7, 0/7 cells showed tLTD) and by AMG9810 (1µM, n=5, 1/5 cells showed tLTD), respectively.

Representative traces are the average of 15 EPSCs during baseline (black traces) and 45 min after STDP protocol (grey traces). Vertical grey dashed line indicates the STDP protocol. Error bars represent sem. *: $p<0.05$; ns: not significant; by t-test, two tailed (b) or one sample t-test (c and d).

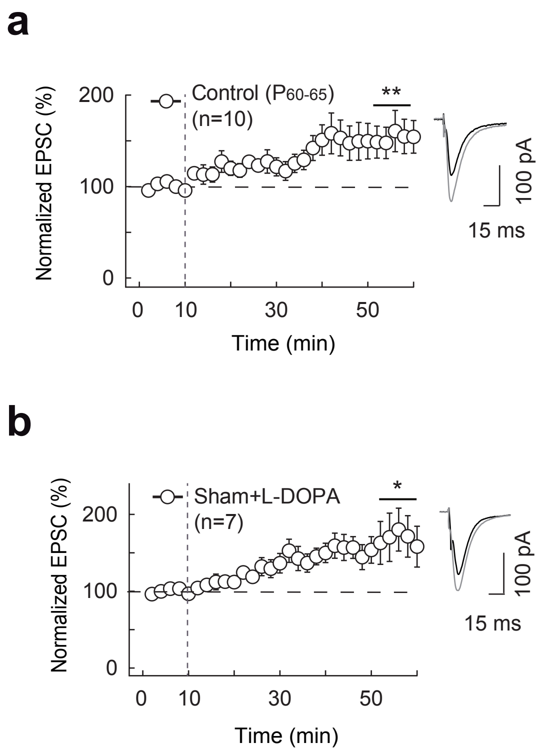

Supplementary Figure 2: Additional control experiments related to Figure 2

(**a**) 10 post-pre pairings induced tLTP in P60-65 rats (n=10, 8/10 cells showed tLTP), *i.e.* rats recorded at the same age than the 6-OHDA-lesioned treated with L-DOPA (and Sham) rats estimated in Figure 2. (**b**) 10 post-pre pairings induced tLTP in Sham-operated rats at P_{35} and treated with L-DOPA from P_{50} to P_{60-65} (n=7, 5/7 cells showed tLTP).

Representative traces are the average of 15 EPSCs during baseline (black traces) and 45 min after STDP protocol (grey traces). Vertical grey dashed line indicates the STDP protocol. Error bars represent sem. *: $p<0.05$; **: $p<0.01$; by one sample t-test (a and b).

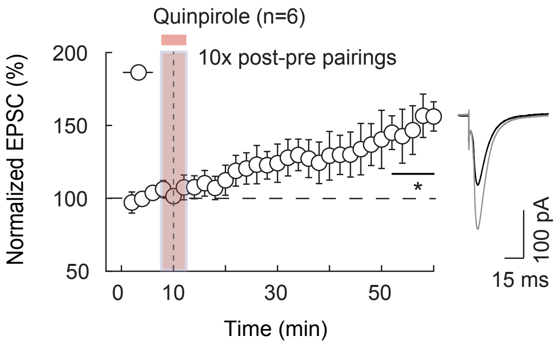

Supplementary Figure 3. D2R activation during STDP pairings did not impact tLTP

Summary of STDP experiments performed with bath-applied quinpirole (10µM, n=6, 6/6 cells showed tLTP), a D2R agonist, during the 10 post-pre pairings.

Representative traces are the average of 15 EPSCs during baseline (black traces) and 45 min after STDP protocol (grey traces). Vertical grey dashed line indicates the STDP protocol. Error bars represent sem. *: $p<0.05$; by one sample t-test.

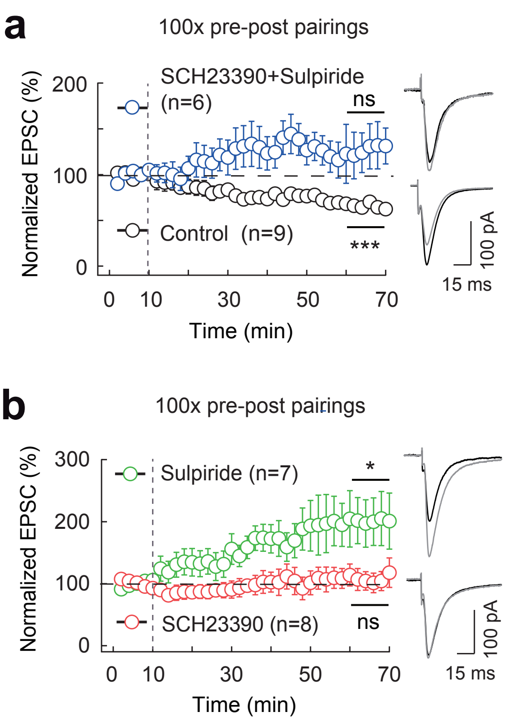

Supplementary Figure 4. eCB-tLTD relies on both D_1R- and D_2R-activation.

(**a**-**b**) tLTD induced with 100 pre-post pairings ($0<\Delta t_{STDP}<+30$ms) is both D1R- and D_2R-activation dependent. Summary of STDP experiments showing that tLTD induced with 100 pre-post pairings is prevented by a mixture of D_1R and D_2R antagonists, SCH23390 (4μM) and sulpiride (10μM) (n=6, 0/6 cells showed tLTD) (**a**) and impaired with either SCH23390 (4μM, n=8, 3/8 cells showed tLTD) or sulpiride (10μM, n=7, 1/7 cells showed tLTD) (**b**).

Representative traces are the average of 15 EPSCs during baseline (black traces) and 45 min after STDP protocol (grey traces). Vertical grey dashed line indicates the STDP protocol. Error bars represent sem. *: $p<0.05$; ***: $p<0.001$; ns: not significant; by one sample t-test (a and b).

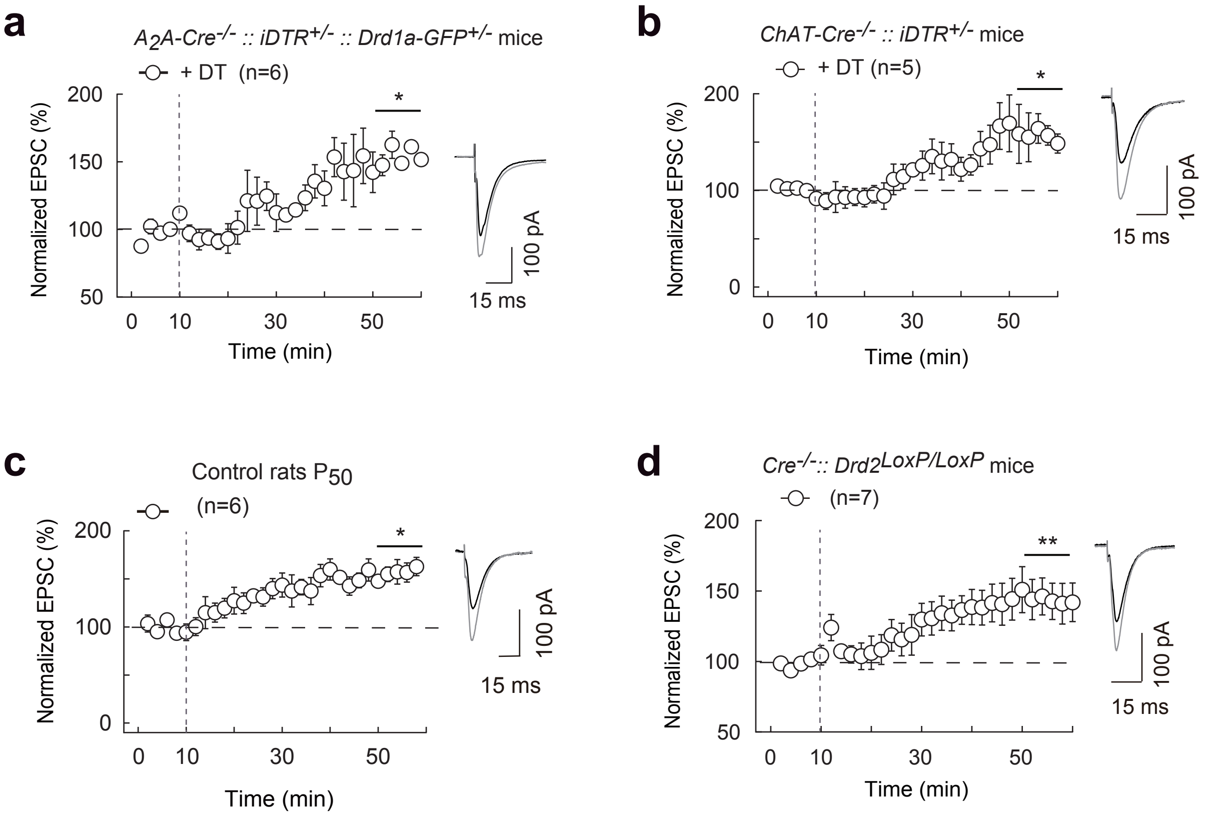

Supplementary Figure 5: tLTP was induced in *A_2A-$Cre^{-/-}$::$iDTR^{+/-}$::$Drd1a$-$GFP^{+/-}$*, *$ChAT$-$Cre^{-/-}$::$iDTR^{+/-}$*, *$Cre^{-/-}$::Drd2LoxP/LoxP* mice and in P_{50} rats.

(**a**) tLTP was also induced in mice lacking DTR with injection of DT (*A_2A-$Cre^{-/-}$::$iDTR^{+/-}$::$Drd1a$-$GFP^{+/-}$*) (n=6, 6/6 cells showed tLTP). (**b**) tLTP was induced with 15 post-pre pairings in *$ChAT$-$Cre^{-/-}$::$iDTR^{+/-}$* mice (n=5, 5/5 cells showed tLTP) with stereotaxic injection of DT. (**c**) 10 post-pre pairings induced potent tLTP in P_{50} rats (n=6, 6/6 cells showed tLTP), *i.e.* rats tested at the same age than rats 6-OHDA-lesioned and Sham estimated in Figure 5c. (**d**) tLTP was observed in mice serving as the floxed gene control (*$Cre^{-/-}$::Drd2LoxP/LoxP* mice) (n=7, 7/7 cells showed tLTP) for the selective D_2R-cKO mice experiments.

Representative traces are the average of 15 EPSCs during baseline (black traces) and 45 min after STDP protocol (grey traces). Vertical grey dashed line indicates the STDP protocol. Error bars represent sem. *: $p<0.05$; **: $p<0.01$; by one sample t-test (a-d).

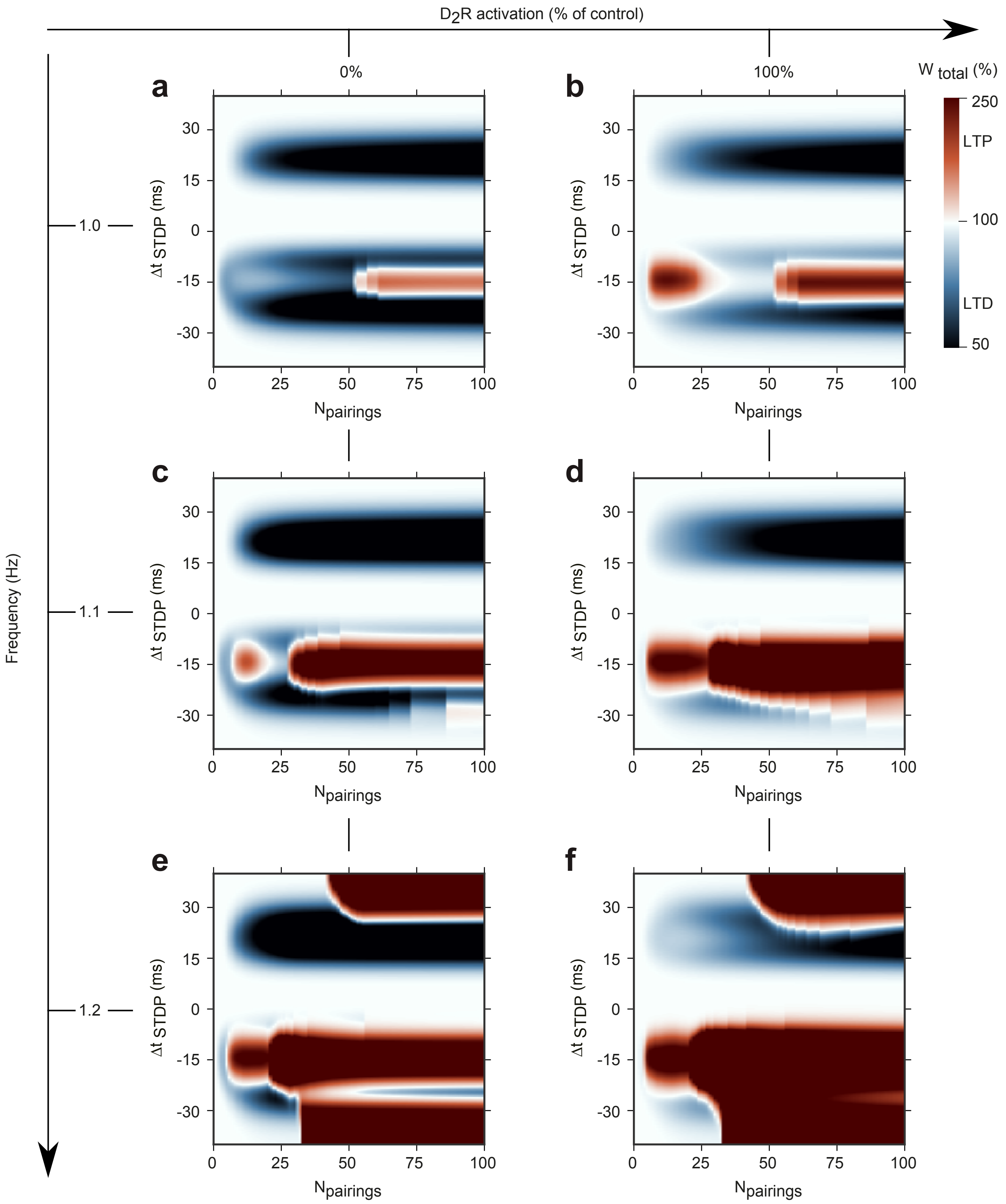

Supplementary Figure 6: Effects of D_2R activation when varying $F_{pairings}$

The model predicts the modulation of plasticity by D_2R activation at various frequencies. The color maps show the dependence of W_{total} on both frequency and $N_{pairings}$ with D_2R activation blocked (**a**, **c** and **e**) or when D_2R is 100% activated (control conditions, **b**, **d** and **f**). (**a**, **c** and **e**) The domain of eCB-tLTP (red) induced with 15 pairings in control conditions (**b**, **d** and **f**), shrinks and shifts to larger frequencies when D_2R are blocked (**a**, **c** and **e**). (**c** and **d**) The domain of NDMAR-tLTP induced with >50 pairings in control conditions (**b**) drastically expands at frequencies >1Hz (**d** and **f**), but displays minor changes when D_2R are blocked, since NMDAR-tLTP is not dependent on presynaptic D_2R (**c**).

All parameters of the model in this figure are the same as in Figure 8b.
